# Dogs understand the role of a human partner in a cooperative task

**DOI:** 10.1038/s41598-024-60772-6

**Published:** 2024-05-03

**Authors:** Juliana Wallner Werneck Mendes, Marie Vindevogel, Ilka van Peer, Mayte Martínez, Giulia Cimarelli, Friederike Range

**Affiliations:** 1 Domestication Lab, Konrad Lorenz Institute of Ethology, University of Veterinary Medicine Vienna, Vienna, Austria; 2 Université de Rennes 1, Rennes, France; 3 HAS University of Applied Sciences, Hertogenbosch, The Netherlands; 4 Language Research Center, Center for Behavioral Neuroscience, Georgia State University, Atlanta, Georgia

**Keywords:** Ecology, Psychology, Zoology

## Abstract

Humans are exceptionally flexible in cooperation, partly due to our ability to recognize the roles of cooperative partners. While some non-human animals understand the need for a partner in such interactions, it is unclear whether they grasp the consequences of their partner’s actions and adjust accordingly. Previous studies utilizing economic games with non-human animals yielded mixed results. We investigated dogs, known for their close cooperation with humans, in a stag hunt game. Dogs could cooperate for better rewards or defect for lower ones, while their human partners would either cooperate, never cooperate, or act randomly. We control for attraction to food, side bias, and local enhancement. Dogs were more likely to coordinate with their partners when it led to better rewards, suggesting that they understood their partner’s actions. By highlighting this cognitive skill in dogs, we advance our knowledge of the intricate mechanisms driving cooperative behavior in non-human animals.

## Introduction

Cooperation can benefit animals in an array of challenges such as solving problems and obtaining desired rewards. However, the choice between cooperative and individual problem-solving involves different risks and outcomes. Accordingly, it is beneficial for individuals to cooperate in specific contexts rather than indiscriminately, rendering decision-making an important aspect of cooperation^1^. Humans show an exceptional flexibility in such choices, partly due our ability to recognize the role of a partner when cooperating^2^.

The string-pulling paradigm has been instrumental for testing whether non-human animals understand the need of a partner in a cooperative context. In this task, two individuals must pull on a rope simultaneously to bring forward a tray containing food; if only one partner pulls, the string detaches, and the food becomes inaccessible. If an individual understands the need of a partner, it is expected that they will wait for a delayed partner to pull the rope. This ability has been shown in elephants^3^, ravens^4^, wolves (both with conspecifics and human partners), and dogs (with humans)^5,6^. The disadvantage of the conventional approach (one table with two ropes) is that if individual “A” chooses to not cooperate, there is nothing else that individual “B” can do. Therefore, this paradigm does not allow to investigate whether and how the choice of a partner influences an individual’s further actions, which is imperative to understand if animals account for the role of the partner.

Economic games allow for simple and often dichotomic decisions of action influenced by the partner’s behavior^7^. The stag hunt game presents options: cooperate (stag) for a high reward or opt for non-cooperation (hare) yielding a lower reward. If, however, one partner chooses the stag and the other one the hare option, the former gets nothing, and the latter gets a low value reward. Thus, there is an inherent risk that if the subject plays stag and the partner does not cooperate (i.e., the partner plays hare), the subject gets no reward. Therefore, in the typical payoff matrix (i.e.: representation of the possible outcomes of a decision) the safe action to play is to choose the lower value but sure reward (hare).

This paradigm has been used to investigate decision-making in humans and non-human primates. Brosnan et al. (2011) found humans, chimpanzees, and capuchin monkeys could learn to play the payoff dominant outcome (both choosing stag), albeit strategies differed (e.g. capuchin monkeys might have used visual matching, while humans and some chimpanzees matched without seeing the partner’s responses)^8^. One way of investigating what mechanisms are being used by subjects is controlling the rate in which the partner chooses stag or hare and testing individuals across different conditions. However, training an individual to make such tailored choices is challenging in non-human animals. To overcome this, Parrish and collaborators (2014) tested humans and rhesus monkeys with a computer algorithm that varied the pattern of stag play between 0 and 100%^9^. Both species were sensitive to the variation in the simulation’s play, learning to adapt their percentage of stag play to match the simulations, but humans did so more than rhesus monkeys, which, instead, showed a stronger bias for playing stag from the beginning of testing. This resulted in both species being equally good in obtaining the payoff dominant stag-stag outcome, although the mechanism they were using to do so was very different: rhesus monkeys might have had a general preference for the better reward, ignoring the choices of their partners, while humans adapted to the partner’s choice in a more flexible way.

Dogs are a promising model to investigate mechanisms for cooperation: they have cooperated with humans for thousands of years^10^, they are capable of following our communicative cues^11^ and producing human-directed communicative signals^12,13^, they are attentive towards humans^14,15^, and they are able to learn socially from us^16,17^. Therefore, testing pet dogs with humans (whom we can instruct on how to behave) is well within dogs’ socio-ecological niche. Recent studies on how dogs cooperate with humans have given us further insights: in Martínez et al. (2024), after being trained to press a button simultaneously with a human partner to obtain a reward, dogs were tested in conditions that varied the temporal contingencies of the human pushing the button. Dogs waited to press in all conditions, showing that they were not reacting merely to the presence of the partner, but being attentive to finer aspects of their actions^18^. However, similarly to the string-pulling task, also in this paradigm, flexibility to a partner’s actions could not be tested, since there were no alternative actions other than pressing the button simultaneously.

To address this aspect, a follow-up study tested pet dogs in an intraspecific stag hunt game using an adaptation of the loose-string apparatus, where dogs could pull the ropes of an apparatus cooperatively to obtain the high value reward, or pull the rope of an individual apparatus to obtain the low value reward^19^. Some dogs were able to coordinate with the choice of their partner, whereas most displayed a strong side bias, with dogs retaining the same chosen side when the positions of the individual and cooperative apparatuses were inverted. Moreover, as with the rhesus monkeys^9^, the choice of the dogs may be explained not by matching their partner but rather by choosing the better reward. Finally, dogs might have performed the same action at the same place after observing the partner doing so, an example of local and/or stimulus enhancement.

In the two above-mentioned studies, as well as in the typical string-pulling studies with dogs^5,6^, dogs received (to different extent) training on the actions they had to perform and the consequences of those actions. Although this shows that dogs can learn to cooperate, it makes it hard to draw conclusions about their understanding of the task or their partners’ role. This can be exemplified by differing findings regarding reciprocity in dogs: Gfrerer & Taborsky (2017) found reciprocity in military dogs^19^, while McGetrick et al. (2024) did not using a sample of pet dogs and a similar set-up but far less training of the reciprocal action^20^. Thus, while these studies show that dogs can be trained to be reciprocal, it remains open if they learn to flexibly adjust their behavior to their partners without explicit training. Moreover, training the consequences of the actions in the stag hunt game renders comparison with primates (including humans) more challenging, as they typically have to figure out the payoff matrix of the game as they play^18,21^.

**Figure 1:**
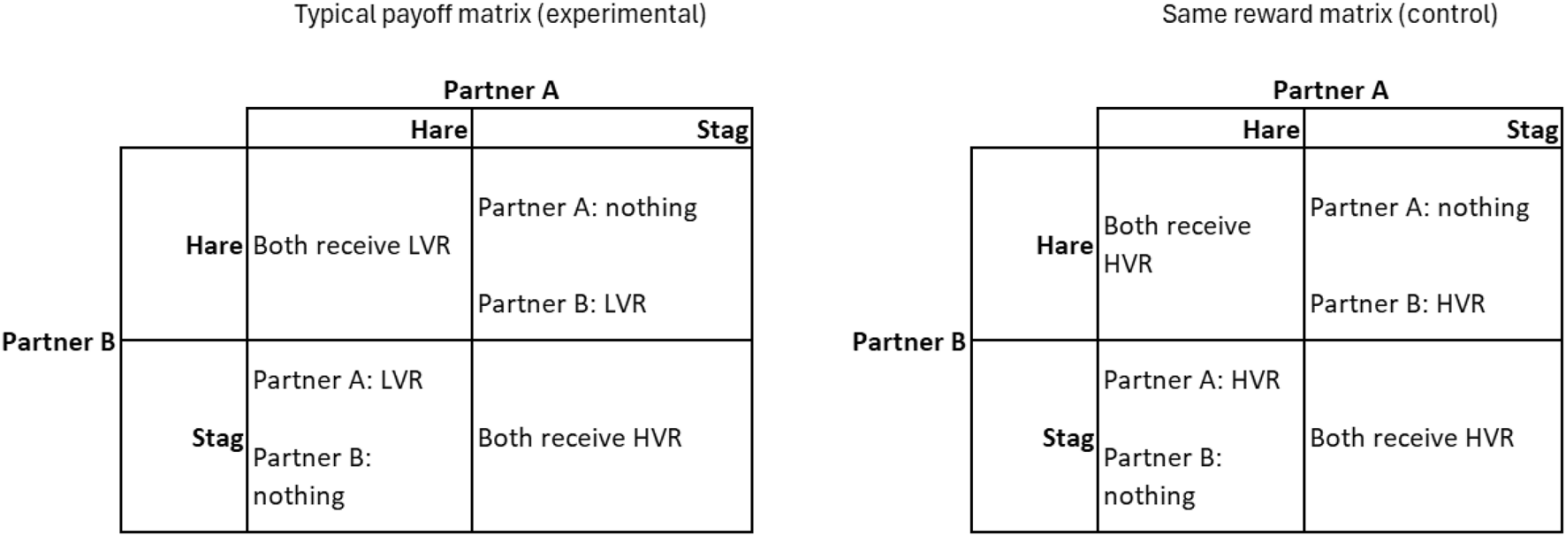
Payoff matrix for the experimental and control group.

In the present study, we investigated whether and to what extent dogs understand the impact of their human partners’ decisions on the outcome of a cooperative stag hunt game and flexibly adjust their own behaviour to their partners’ actions. Moreover, we accounted for the influence of side bias, stimulus enhancement, local enhancement, and attraction to the high value reward. An “experimental group” was presented with the cooperative and the individual trays with the typical payoff matrix (see Fig. 1). Conversely, a “same reward control group” was also presented with the cooperative and the individual trays, but in this case, both had the same high value reward. These two groups allowed us to analyse whether attraction to the high-value reward can explain the decisions made by the dogs. Dogs observed the partners making one of the two choices: hare, represented by the individual table, or stag, represented by the cooperative table. To further observe if dogs made consistent choices and adjust to the actions of the partner, we grouped sets of trials in three different conditions: the partner was always willing to cooperate (payoff dominant condition), partner always worked individually (risk-averse condition), and partner chose randomly to cooperate or not, with approximately 50% chance of cooperating (random condition). As the objective was to test for flexibility in adjusting to the choices of the partner, we used a within-subject design and refrained from extensive training as is usual in such experiments.

We asked two main questions: (1) do group (experimental/group representing the pay-off matrix), trial, and the choice of the partner (human choosing stag or hare, regardless of condition) affect dogs’ choices? The analysis associated to this question provided information on dogs’ decision making and attentiveness to the partner’s choice at the trial level, independent of condition; (2) do group, trial, and condition affect the probability of *matching* the partner’s choice (i.e. dogs making the same choice as the human partner)? This approach allowed us to test dogs’ ability or intention (or lack thereof) to match consistently with the choice of the partner in different conditions. Both analyses also provided information on whether the animals changed their decision making processes over trials. Moreover, we designed the apparatuses to reduce (and control for) the possibility that side bias as well as stimulus or local enhancement would drive the results.

Table 1 shows our hypotheses and the respective predictions, as well as the model used to test each (see methods).

**Table 1:** Hypotheses and predictions tested in the study with the according models.

Hypothesis	Prediction	Tested with
1) Dogs (learn to) understand that the choice of their partners affects them and act accordingly	1a) Dogs in the experimental group will be overall more likely to choose stag when the partner chose stag than when the partner chose hare, as this optimizes their outcome	Model 1
	1b) Dogs in the control group will be more likely to choose hare regardless of the partner’s choice, as choosing hare entails less effort and yields the same reward	Model 1
	1c) Condition (partner always choosing stag, always choosing hare, or choosing randomly) will not affect the likelihood of dogs in the experimental group coordinating with their owners, thus showing flexibility to adjust to the partner’s choices	Model 2
	1d) In the control group, we would see more matching in the condition risk-averse (owner always choosing hare) than in the conditions random (owner choosing hare 50% of the time) and payoff dominant (owner always choosing stag)	Model 2
2) Dogs do not (learn to) understand that the choice of their partners affects them and are guided by preference for the high reward food	2a) Dogs in the experimental group would always be more likely to choose stag than hare, as they would go to where the preferred food is	Model 1
	2b) Dogs in the control group will be more likely to choose hare regardless of the partner’s choice, as choosing hare entails less effort and yields the same reward	Model 1
	2c) Dogs in the experimental group would be more likely to match their partners’ choice in the condition payoff dominant than in the conditions random and risk-averse	Model 2
	2d) In the control group, we would see more matching in the condition risk-averse (owner always choosing hare) than in the conditions random (owner choosing hare 50% of the time) and payoff dominant (owner always choosing stag)	Model 2
3) Dogs do not (learn to) understand that the choice of their partners affects them and preferably follow the owner	3a) Dogs in both the experimental and in the control group to be more likely to choose stag when the partner chose stag, and choose hare when the partner chose hare, as they would go to the same place where the human is	Model 1
	3b) Condition would not affect the likelihood of dogs coordinating with the partner neither in the experimental nor in the control group	Model 2

## Methods

### Subjects

Dog owners were recruited through the Clever Dog Lab database and participated voluntarily. We tested 19 dogs between 1 and 12 years of age. They had been living with their owners for at least 1 year and were from various breeds or mixed breeds (see supplementary material for details). Ten dogs were assigned to the experimental group and nine to the control group, as described below.

### Procedure

We used the economic game stag hunt, where two individuals must choose between one of two options, stag or hare. The human made the initial choice based on predetermined conditions (see "experimental conditions" below), and the dog’s choice followed afterward. If both participants chose "hare", they both received a low value reward. If they both cooperated by choosing "stag," they both received a high value reward. However, if one tried to cooperate by choosing "stag" while the other defected by choosing "hare," the cooperator received nothing, and the defector received a low-value reward (see Fig. 1 for the matrix). In our setup, "stag" represented a high value reward (HVR) achievable only through cooperative actions by the dog and owner, while "hare" represented an apparatus the dog could solve independently, resulting in a low value reward (LVR).

### Experimental area and apparatus

**Figure 2:**
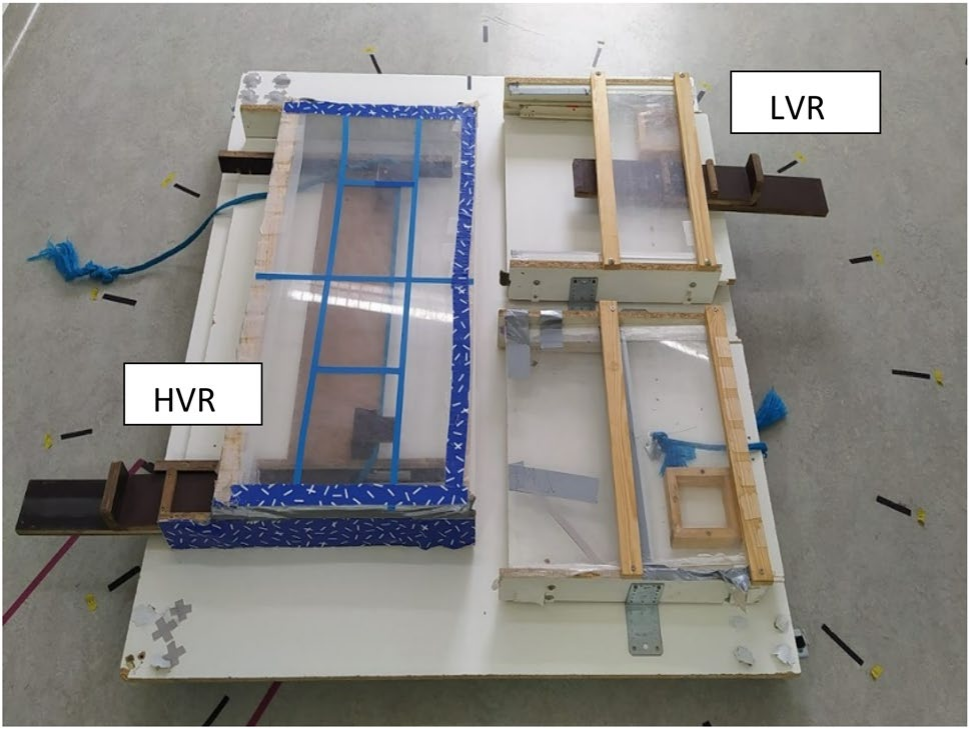
Experimental apparatuses. On the left size is the cooperative table where the high value reward (HVR) was placed. On the right, the two individual tables where the low value reward (LVR) was placed.

The experiment took place in a room of 3.3 m × 6.05 m in the Clever Dog Lab, University of Veterinary Medicine, Vienna. Part of the room (2 m × 1.5 m) was separated from the rest by an opaque partition that prevented the dog from seeing the baiting of the apparatus. The apparatus consisted of a rotating platform (1.8 m × 1.5 m) with the “cooperative table” (1 m × 60 cm) on one side and the “individual tables” (50 cm × 60 cm each) on the other side (Figure 2). On the individual side, one of the tables had a rope that could be pulled, bringing the platform forward and making the food accessible. The other table had a drawer with a handle that could be pulled, having the same effect. The cooperative table had both a rope and a drawer. To access food on this side, both the rope and drawer needed to be pulled simultaneously. If only one action was performed at a time on the cooperative side, the platform remained in place. To visually distinguish the cooperative table for the dogs, it was colored blue, while the individual tables were white. Additionally, the floor was marked with circular tape, with black tape segments placed every 20 degrees to provide visual alignment reference points with a corresponding black tape on the platform (upper middle part, Figure 2).

### Training

To avoid experience with the contingencies of the task and the outcome of the partner’s choices (see Introduction), dogs were trained to perform the action (pulling the rope or the drawer) on a separate apparatus and with a different food reward (see “dog training” in supplementary information). The choice of having two different actions was made to avoid that dogs would match the partner’s choice purely based on stimulus enhancement—dogs performing the same action they saw their partner perform.

### Testing: experimental group

Each subject participated in six sessions of 30 trials each. Within a test session, a break of 5–10 min was made after 15 trials. Each session was conducted on a different day, totalizing six days for each dog. Before the session, the experimenter randomized the position in which the apparatus would be in each trial considering the possible 18 markings on the floor separated by 20° each. The experiment started with the owner holding the dog behind the opaque partition, so the dog could not see the apparatus. After positioning the apparatus, the experimenter baited the tables, putting the HVR on the cooperative and the LVR on the individual table. Then, the experimenter went behind the partition, held the dog, and said “okay” to the owner. The owner moved to their position next to the apparatus (according to the conditions described below) and held the rope (for the dogs that were trained to pull the drawer) or touched the handle of the drawer (for the dogs that were trained to pull the rope). The dog was then released by the experimenter saying “okay”. If the dog touched the handle of a drawer with a paw or picked up a rope with its mouth (according to the action they were trained to do), they were considered to have made a choice. If the dog chose the cooperative side while the owner was there, the owner coordinated their behaviour with the dog, making the food accessible. If the dog chose the individual side, the owner did not do anything. After the dog ate the food or made a choice that did not allow it to get food, (i.e., choosing stag when the owner was on the hare side), they were called back to the initial position and were not allowed to make a second choice. If the dog did not make a choice within 1 min, the trial was terminated. The experimenter then rotated the apparatus to the new position and re-baited the table when necessary, while the dog was behind the opaque partition. This was repeated in every trial (see Video 1 in SI for an example of the procedure).

In the start of the test, the dog had no knowledge of the functionality of the cooperative table or what food reward could be found on each side. Therefore, they had to learn the contingencies by trial and error.

### Testing: control group

The control group were subjected to the same experimental procedure, but they had HVR both on the cooperative side and on the individual side.

### Experimental conditions

**Figure 3:**
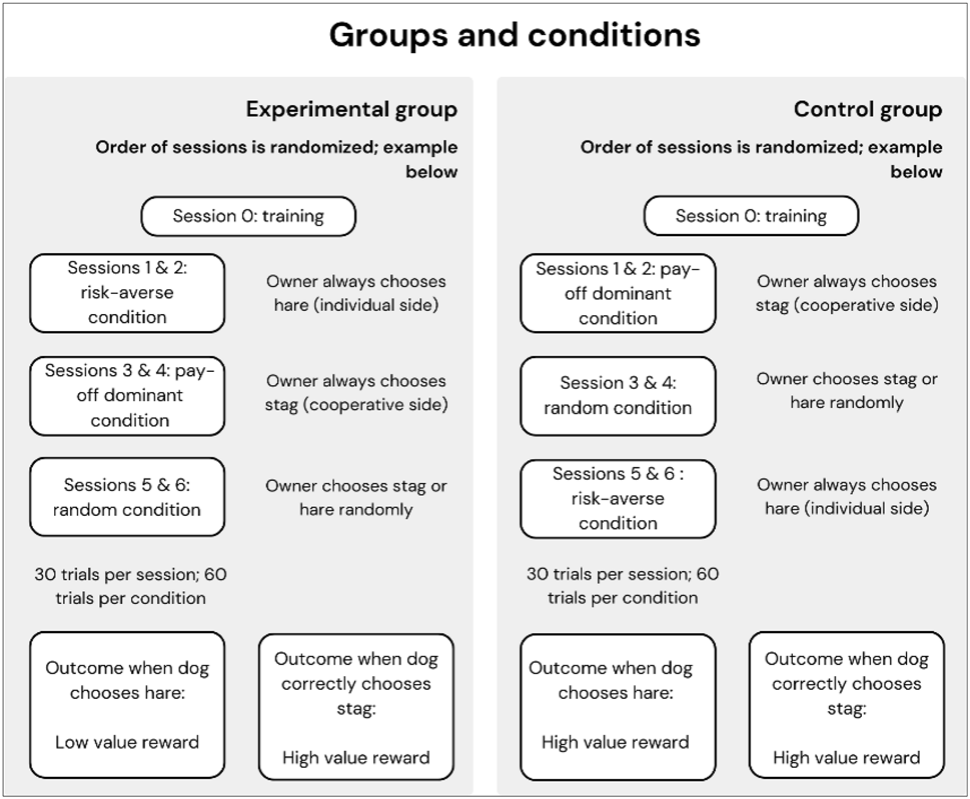
Illustration of the experimental design. Note that the order in which conditions were presented was counterbalanced across subjects, and the orders presented are an example.

The owner behaved according to three different conditions, which were consistent across two sessions (totalizing 60 trials per condition): “payoff dominant” condition (always chose stag), “risk averse” condition (always chose hare), or a “random” condition where the human chose randomly and there was no stable “solution” for the animals (that is, the subjects needed to adjust their choice in each trial, depending on what the partner did). For the random condition, the experimenter randomized the choices and informed the owner about where to go by pointing of the respective choice on a piece of paper. Subjects were confronted with each condition in a semi-randomized order, counterbalanced across subjects. The two sessions of the same condition always happened sequentially (see Fig. 3).

### Ethics

All methods were performed in accordance with the relevant guidelines and regulations. All procedures used in the present study were approved by the institutional Ethics and Animal Welfare Committee at the University of Veterinary Medicine Vienna, in accordance with Good Scientific Practice guidelines and national legislation (protocol number ETK-165/10/2021). Additionally, the study is reported in accordance with the ARRIVE guidelines. Written informed consent was obtained from the owners for their participation with their animals in the study and for publication of images and data included in this article. The Ethics Committee at the Medical University of Vienna deemed that approval for human participation was not required.

### Coding

Videos were recorded by three different cameras in the Clever Dog Lab room. They were then coded on Solomon Coder beta 09.08.02 (copyright 2019 by András Péter, developed at ELTE TTK Department of Ethology, Budapest, Hungary). The following variables and corresponding definitions are reported in Table 2.

**Table 2:** Ethogram of main coded behaviors.

Behavior	Definition	Variable type
Partner choice: hare	The owner grabs the rope or the drawer with his/her hand in the individual table	Binary
Partner choice: stag	The owner grabs the rope or the drawer with his/her hand in the cooperative table	Binary
Subject choice: hare	The dog touches the rope with its mouth or drawer with its paw in the individual table	Binary
Subject choice: stag	The dog touches the rope with its mouth or drawer with its paw in the cooperative table	Binary
Success	Upon completion of the action, the dog obtains the food in the chosen table	Binary
Match	The subject’s choice is hare when the partner’s choice is hare OR the subject’s choice is stag when the partner’s choice is stag	Binary
Side: right	Drawing an imaginary line exactly in the middle of the room, the dog chooses the table on the right side of the line	Binary
Side: left	Drawing an imaginary line exactly in the middle of the room, the dog chooses the table on the left side of the line	Binary
Distance: close	The dog chooses the table closest to their starting point	Binary
Distance: far	The dog chooses the table furthest to their starting point	Binary

Partner choice was predefined and therefore not coded in the videos.

A second person coded 20% of the videos and the interobserver reliability was appropriate for every variable (Cohen’s kappa > 0.75).

### Statistical analysis

Dogs made a choice in 96% of the trials. Choice of the partner (human choosing stag or hare, from here on “partner’s choice”) and condition are highly correlated but do not portray the same information: in the payoff dominant condition, the partner always chose stag; in the risk-averse condition, the partner always chose hare; however, in the random condition, the partner chose both hare and stag (in different trials). We ran two models, the first one investigating whether the choice of the partner (human choosing stag or hare), group, and trial affected the choice of the subject (dog choosing stag or hare, from here on “subject’s choice”). This model provided information on dogs’ decision making and attentiveness to the partner’s choice on the trial level, independent of condition. The second model investigated whether group, condition, and trial affect the probability of *matching* (dog making the same choice as the human partner). This model portrayed information about dogs’ ability (or lack thereof) to coordinate consistently with the choice of the partner in different conditions. We fitted both models in R (Version 4.1.2), using the function glmer of the package lme4 (version 1.1–27.1). The sample analyzed for these models comprised a total of 3390 observations of 19 dog–human pairs. Confidence intervals and model stability for both models are presented in SI (SI Tables 1 and 2). We additionally made plots of individual dogs’ choices (SI Fig. 3).

### Model 1

To estimate the effects of group and the partner’s choice on the subject’s choice, we used a generalized linear mixed model (GLMM) with binomial distribution. As we anticipated that the influence of the partner’s choice might be more pronounced in the experimental group and that a possible learning effect would be different according to group and partner’s choice, we included the interaction of group, partner’s choice, and trial. To control for potential carry-over effects across sessions, we included session in the model. To control for a possible side or distance bias, we included chosen side and distance in the model. Distance (close/far) was included because, as the apparatus rotated, there was always a table that was closer to the dog’s starting point. Lastly, to account for possible differences in age of the dogs, we included age in the model as a control variable. To avoid a model being overconfident with regard to the precision of fixed effects estimates and to keep type I error rate at the nominal level of 5%, we included all theoretically identifiable random slopes^22^. More precisely, we included the random slopes of session order, chosen side, distance, and the interaction between trial and partner choice. Prior to fitting the model, we z-transformed trial, session, and age to a mean of zero and a standard deviation of one, and dummy coded the categorical variables—condition, chosen side, and distance—and centered the dummy coded variables at risk-averse, left, and close, respectively, before including them in the random slopes^23^. As an overall test of the effect of the fixed effects and to avoid “cryptic multiple testing”^24^, we compared the full model as described above with a null model lacking the fixed effects but being otherwise identical. We tested the effect of individual fixed effects by comparing the full model with reduced models lacking them one at a time. To simplify the model and ease the interpretation of the main effects, we removed non-significant interactions (one at a time) until we obtained a model consisting in only main effects and significant interactions.

### Model 2

To estimate the effects of group and condition on whether the subject matched their partner’s choice, we used a GLMM with binomial distribution. We included the interaction of group, condition, and trial. For the same reasons as the previous model, we included session, chosen side, distance, and age as control predictors, as well as the random slopes of session order, trial, side, distance, and the interaction of trial and condition. Prior to fitting the model, we z-transformed trial, session, and age to a mean of zero and a standard deviation of one, and dummy coded the categorical variables—condition, chosen side, and distance—and centered the dummy coded variables at risk-averse, left, and close, respectively, before including them in the random slopes. We compared the full model as described above with a null model lacking the fixed effects but being otherwise identical. We tested the effect of individual fixed effects by comparing the full model with reduced models lacking them one at a time^22^. For these tests as well as the full-null model comparison we utilized a likelihood ratio test^25^. To simplify the model and ease the interpretation of the main effects, we removed non-significant interactions (one at a time) until we obtained a model consisting in only main effects and significant interactions. We further investigated the pair-wise comparisons in each condition using the function emmeans, and performed a post hoc binomial test to investigate whether the likelihood of matching was above chance across groups and conditions.

### Writing

We used OpenAI, ChatGPT (August 24th 2023 version) to verify and improve readability of the introduction and the discussion sections. The prompt used was “Improve the readability of this scientific manuscript without changing any content, keeping a similar size and style”. The generated version was then compared to the original version, and the authors edited specific sentences in the original version when appropriate. Approximately 5% of the introduction was altered based on ChatGPT generated text, and approximately 10% of the discussion.

## Results

### Effect of partner’s choice on subject’s choice (Model 1)

The full-null model comparison revealed that the predictors had a significant effect on the dogs’ choices (χ2 = 24.70, df = 6, *P* < 0.001). In particular, dogs in the experimental group were overall more likely to choose stag than dogs in the control group (χ2 = 6.54, df = 1, *P* < 0.01; Table 3). Dogs were also more likely to choose stag when the owner chose stag (χ2 = 17.24, df = 1, *P* < 0.001; Table 3). Dogs significantly changed their choices across trials (X^2^ = 4.135, df = 1, *P* = 0.042; Table 3). There was not a significant interaction between group, partner choice, and trial.

**Table 3:** Results of subject’s choice model (estimates, standard errors and significance tests).

Term	Estimate	SE	X^2^	df	*P*
Intercept	− 2.537	0.436	–	–	–
**Group (1)**	1.303	0.482	6.543	1	0.011
**Partner choice (1)**	1.049	0.259	17.244	1	< 0.001
**Trial (2)**	− 0.179	0.089	4.135	1	0.042
Session (2)	− 0.701	0.249	13.806	1	< 0.001
Age (2)	− 0.579	0.257	11.311	1	< 0.001
Side (1)	–	–	3.319	2	0.190
Distance (1)	0.039	0.123	0.095	1	0.757

(1) Reference level for categorical variables are: group (control), partner choice (hare), side (left), distance (close).

(2) z-transformed to a mean of zero and a standard deviation (sd) of one.

**Figure 4:**
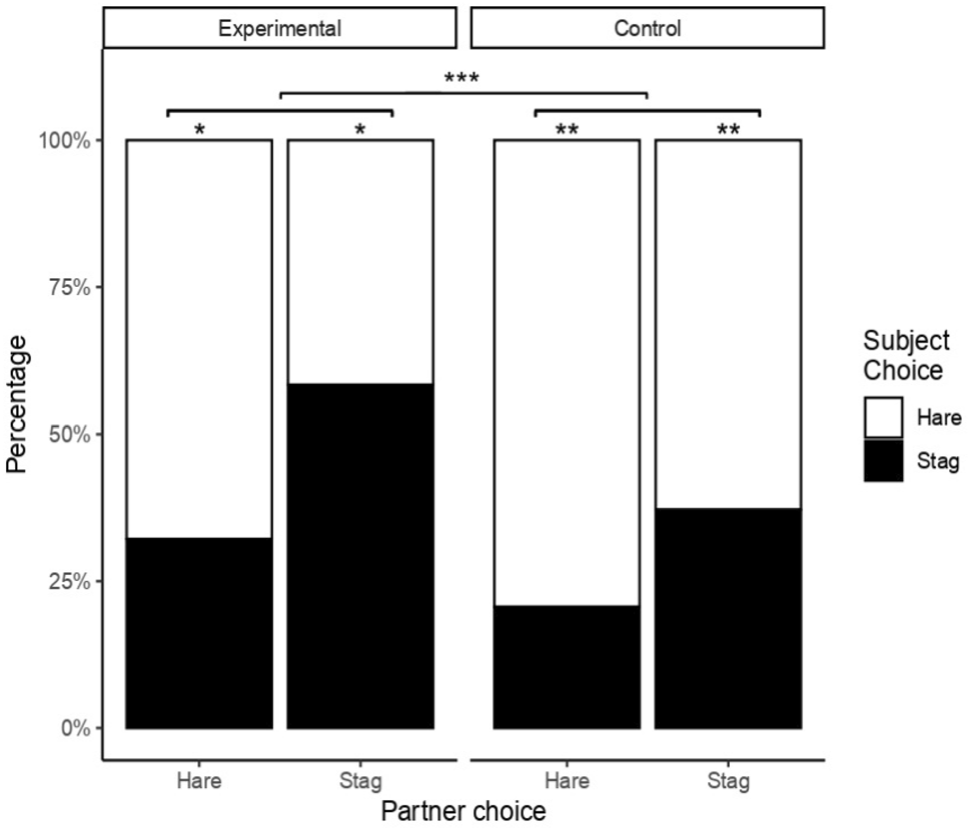
Bar charts showing the frequency of the dog’s choice according to the partner’s choice. The x axis shows the partner’s choice (hare or stag). The white bar portion of the bar shows the frequency in which the dogs chose hare, and the black portion of the bar shows the frequency in which the dogs chose stag.

In the experimental group, when the human chose stag, dogs chose stag 58% of the time, while they chose hare 68% of the times if the human chose hare. In the control group, when the partner chose stag, dogs chose stag 37% of the times, while when the human chose hare, the dogs chose hare 79% of the times (Fig. 4).

### Effect of group and condition on matching (Model 2)

**Figure 5:**
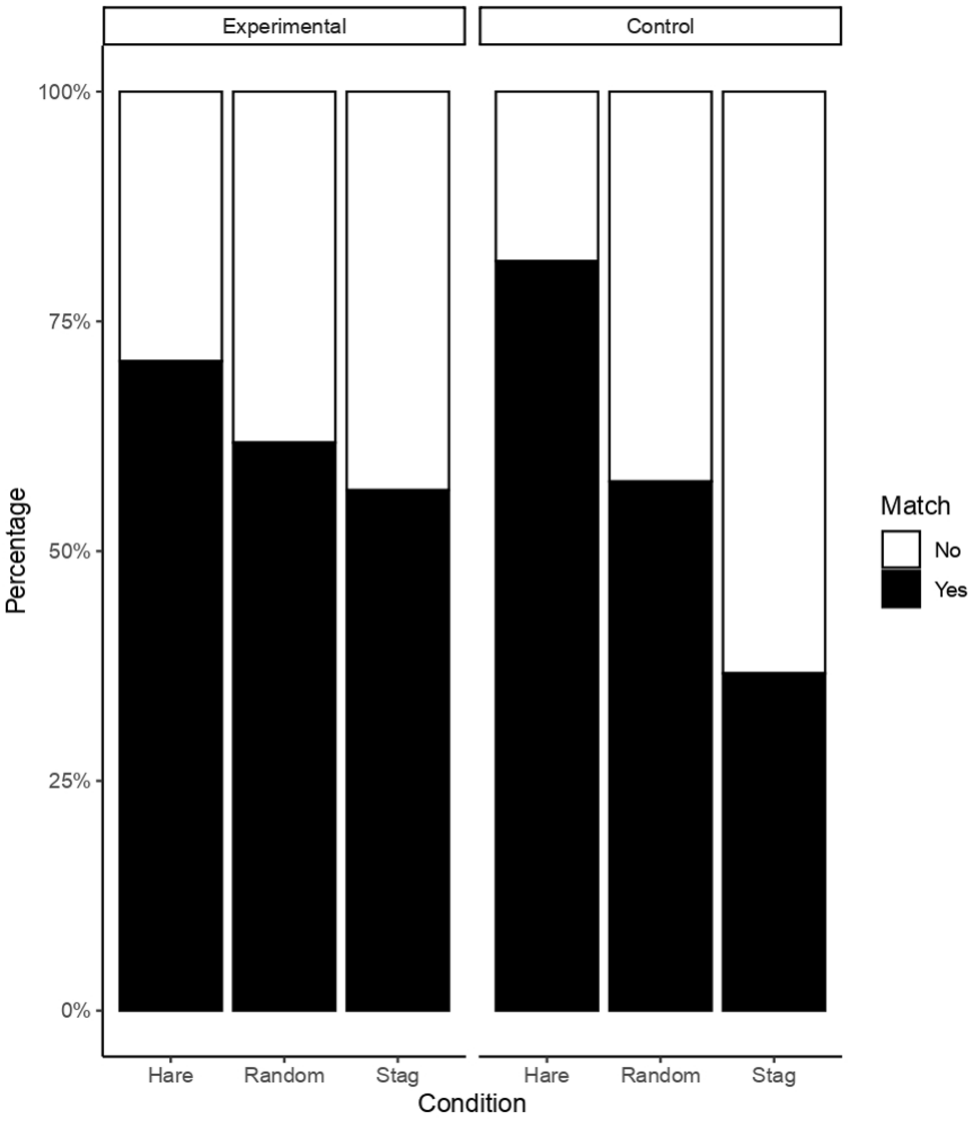
Bar charts showing the percentage in which dogs matched the choice of the partner (i.e.: dog chose hare when partner chose hare, and dog chose stag when partner chose stag) according to condition, in control and experimental group.

The full model differed from the null model (χ2 = 27.86, df = 10, *P* = 0 = 0.002), with the interaction between group and condition being significant (χ2 = 8.698, df = 2, *P* = 0.0129; Table 4). In the experimental group, the likelihood of matching was not significantly different across conditions (56% in the payoff dominant condition, 67% in the risk-averse condition, and 60% in the random condition; Fig. 5, Table 5). In the control group, dogs’ likelihood of matching was the highest in the risk-averse condition and tended to be the lowest in the payoff dominant condition (80% in the risk-averse condition, 56% in random, and 36% in the payoff dominant; Fig. 5). The difference was significant between risk-averse and random and tended towards significance between risk-averse and payoff-dominant (Table 5). A binomial test showed that dogs matched their partners’ choices differently from chance in all groups and conditions (Table 6).

**Table 4:** Results of model for matching choices (estimates, standard errors and significance tests).

Term	Estimate	SE	X^2^	df	*P*
Intercept	1.884	0.424	–	–	–
Trial (1)	0.055	0.084	0.433	1	0.510
Session (1)	− 0.026	0.094	0.076	1	0.783
Age (1)	0.257	0.117	4.099	1	0.043
Side (2)	0.03	0.146	4.508	2	0.105
Distance (2)	–	–	0.041	1	0.840
Group*Condition (2)	–	–	8.698	2	0.013

(1) z-transformed to a mean of zero and a standard deviation (sd) of one.

(2) Reference level for categorical variables are: group (control), condition (risk-averse), side (left), distance (close).

**Table 5:** Pair-wise comparison of the effect of condition in the experimental and in the control group.

Contrast	Estimate	SE	z.ratio	*P* value
*Control*				
Risk-averse/random	1.242	0.363	3.426	0.002
Risk-averse/pay-off dominant	2.254	0.978	2.305	0.055
Random/pay-off dominant	1.012	0.794	1.275	0.409
*Experimental*				
Risk-averse/random	0.0684	0.38	0.18	0.9823
Risk-averse/Pay-off dominant	0.1495	1.022	0.146	0.9883
Random/pay-off dominant	0.0811	0.84	0.097	0.9949

**Table 6:** Results of the binomial test across groups and condition.

Group	Condition	Min CI	Max CI	*P*
Experimental	Payoff dominant	0.519	0.600	0.003
	Risk-averse	0.631	0.708	< 0.001
	Random	0.566	0.646	< 0.001
Control	Payoff dominant	0.322	0.405	< 0.001
	Risk-averse	0.767	0.838	< 0.001
	Random	0.514	0.600	0.009

## Discussion

Our findings support our main hypothesis that dogs learn to comprehend their partner’s role in the cooperative task. Dogs in the experimental group were generally more likely to choose stag than dogs in the control group, and dogs were more likely to choose stag when the partner was choosing stag than when the partner was choosing hare (H1, predictions A and B, Table 1). Condition did not influence dogs in the experimental group, who consistently matched their owner’s choice, optimizing their outcomes (H1 prediction C, Table 1). Moreover, the control group dogs were more likely to match their owner’s choice in the risk-averse condition than in the random condition, and tended to match more in risk-averse than in the payoff-dominant condition (H1, prediction D, Table 1).

Our design accounted for potential confounding factors—side bias, stimulus enhancement, local enhancement, and attraction to the high value reward—allowing us to rule out alternative explanations. For instance, dogs were not only following the humans (i.e., local enhancement), since, in the control group, they did not coordinate with their owners indiscriminately, but rather maximized their outcomes by choosing the risk-averse option independent of their partner’s choice. This is in line with a previous study using a different experimental set-up^18^, where it was shown that dogs do not merely react to their human partner’s presence, but take their actions into consideration. In that study, after training dogs to press a button at the same time as the owner for a reward, dogs were able to coordinate actions even when the human partner arrived but waited as long as 9 s to press the button, showing that they were paying attention to their action and not only pressing when the human was next to the button.

In the present study, dogs were also not exclusively attracted to the high value reward, as dogs in the experimental group did not choose stag indiscriminately. Interestingly, these results are similar to the pattern of distributing responses to each alternative in proportions that are equivalent to their payoff structure seen in humans^26^. Dogs differed from other non-human animals, which exhibited a bias towards the highest paying option^9,27^. For instance, rhesus macaques had a stronger bias to play stag (possibly attracted to the high value reward) independently of their partner’s choice^9^. Their bias towards stag might be the reason why both partners chose the payoff dominant option, and even when they did not have visual access to their partner’s choices^28^. Other species, such as capuchin monkeys, are not able to maintain coordination once they cannot see their partner´s choice, which suggest that they coordinate by matching their partner´s choice^8,28^. This matching strategy was also present in dogs in a previous study with the stag hunt in which dogs could always see their partner’s choices^19^. The authors found that dogs adapted their choices to match their partner´s choice, but mechanisms such as side bias influenced dogs’ performance, an aspect we could rule out in the present study thanks to the circular design of the apparatus. In the present study, we also used a version of the task in which partner´s actions were visible. Thus, visual matching was likely an important aspect in dogs’ decision-making and coordinating, as shown in previous studies. For example, in a string-pulling task, less experienced dogs gazed more at the partner, and there was an overall increase of looking at the partner across sessions, indicating dogs learned to pay attention to the partner’s actions over trials^6^.

Even with intermixed conditions and no training on the contingencies of the task, dogs were able to act in accordance with an understanding of the role of the partner and the consequences of their actions. However, this does not mean that lower level mechanisms did not play a role. For instance, dogs in the control group did cooperate on stag with humans 36% of the time, potentially showing that dogs were attracted to their human partner to some extent. This is in line with findings showing that dogs are more likely to choose a smaller quantity of food when their owners showed a preference for it^29^, potentially showing dogs’ reduced independent acting and problem-solving in the presence of a human. Moreover, dogs in the experimental group chose stag 33% of the time when the partner was always choosing hare, a possible result from partial attraction to the preferred food. Alternatively, it is possible that the absence of a perfect match with the theoretical predictions was due to dogs exploring the contingencies of the game, as they were given no previous experience on how and where they could obtain each reward. The same reason could explain why dogs in the experimental group cooperated with their owners in the payoff dominant condition only in 56% of the time, despite the fact that they could have received the high value reward in every trial if they would have matched their partner’s choice reliably. However, we would then have expected a learning effect across trials. Although dogs did change their choices across trials, as shown by model 1, the likelihood of matching their owner’s choice did not significantly improve across trials, as shown by model 2. It is possible that 60 trials per condition were not enough for the dogs to learn the contingencies reliably, especially given that we intermixed conditions across. Therefore, it is important to note the possibility that the dogs’ choices might have further improved with further trials due to further learning. Finding out the payoff matrix of the stag hunt game without previous exposure has proven itself also a hard task in primates, including humans: when not given instructions in the stag hunt, only 19% of human pairs eventually cooperated on stag^8^. While it is important to note that Brosnan and collaborators analyzed the number of *pairs* that coordinated rather than number of trials in which coordination was achieved, the results demonstrate the challenge of finding the highest payoff matrix. Another non-exclusive explanation to dogs’ high rate of choosing hare is that some dogs were partially using a risk-averse strategy themselves, maintaining the hare choice as it always guaranteed some reward. Visual inspection of individual dogs’ choices (SI Fig. 3) shows this might be the case for at least two dogs in the experimental group (Fanny and Sepp). Importantly, due to the different order of conditions, although counter-balanced, dogs might have used different strategies in different conditions, as it has been shown that dogs use different strategies depending on tasks and difficulty levels^30^.

Explicitly testing for the mechanisms behind cooperation, our results indicate that dogs understood the role of their partner and the consequences of their actions when cooperating rather than being exclusively driven by side bias, stimulus enhancement, local enhancement, and/or attraction to the high value reward. Importantly, dogs went through minimal training and had no exposure to the contingencies of the task and the payoff matrix before the test. Therefore, dogs were able to learn the results of different (potentially) cooperative interactions by trial and error. It is important to point out that the use of different set-ups to test different species (e.g. token exchange or touch screen for primates, rope-pulling or buzzer pressing for dogs), although aimed at maximizing the ecological validity of the paradigm used, renders a direct comparison between studies limited. We suggest that to properly highlight differences between species in cooperative decision-making would require direct comparative work^8,31^. Moreover, dogs have a differential relationship with humans^15^ that possibly influences the results with some dogs possibly matching their owner’s choices more often than others. Future studies should investigate intraspecific cooperation, the influence of visibility of the partner’s actions and further manipulate the dogs’ choices to tease apart what strategies they use to make decisions. Overall, we show that a non-human animal can grasp the consequences of their partner’s actions and adjust accordingly in a cooperative interaction.

### Supplementary Information

Supplementary Video 1.

Supplementary Information 1.

## Data Availability

The raw data and the script describing the statistical analysis are available in the repository Phaidra, under the identifiers https://phaidra.vetmeduni.ac.at/o:2480 and https://phaidra.vetmeduni.ac.at/o:2123, respectively.
