# Supplementary material for: Dogs understand the role of a human partner in a cooperative task
Source: Sci Rep. 2024 May 3;14:10179. doi: 10.1038/s41598-024-60772-6 (PMC11068782; doi:10.1038/s41598-024-60772-6)
Supplement: Supplementary file 2 — Supplementary Information 1. [file 41598_2024_60772_MOESM2_ESM.docx]

**Supplemental information**

**Dog training**

Dogs were trained on a separate apparatus that had the same mechanics as the experimental apparatus but looked different and was stationary. This approach ensured minimal training in regard to the actual experimental set-up while giving the dogs the knowledge on how perform the action to obtain a reward. The food used for the training was different than the LVR and HVR used in the test (i.e.: we used cheese for the training for dogs that had sausage as the HVR, and sausage for dogs that had cheese as the HVR; see below for more details regarding the food used during testing).

Dogs on both the experimental and the control group were trained to use the drawer, while the human partner was always pulling the rope. This was the case because pilot trials showed that if dogs were trained on the rope and the human was responsible for the drawer, dogs were more likely to spontaneously use the drawer and “steal” the position of the human. Such behaviour would have added contingencies to the experimental set-up that are not typically a part of the stag hunt game. Two dogs, however, did not learn to pull the drawer and were trained to pull a rope with their mouths instead.

For the training with the drawer, while the dog was close to the apparatus, the experimenter showed them food and put it on the plate. Then, whenever the dog touched the handle with the paw, they received a piece of food. After five trials in which the dog only needed to touch the handle, the experimenter waited until the dog made any pulling motion before rewarding (e.g. shaping). This process continued until the dog completely pulled the drawer out and ate the food from the plate. For the two dogs that were trained on the rope-pulling apparatus, while the dog was close to the apparatus, the experimenter showed them the food and put it on the plate. Then, when the dog grabbed the rope with its mouth, they received a piece of food. After five trials in which the dog only needed to grab the rope, the experimenter waited until the dog pulled it slightly before rewarding (e.g. shaping). This process continued until the dog pulled the plate completely out and ate the food. In both cases, the dog was considered trained when they performed the action and ate the food 10 times in a row without encouragement of the trainer. The owner was present in the room where the training was taking place, eventually encouraging the dog to look for the food when the dog was not interacting with the apparatus.

**
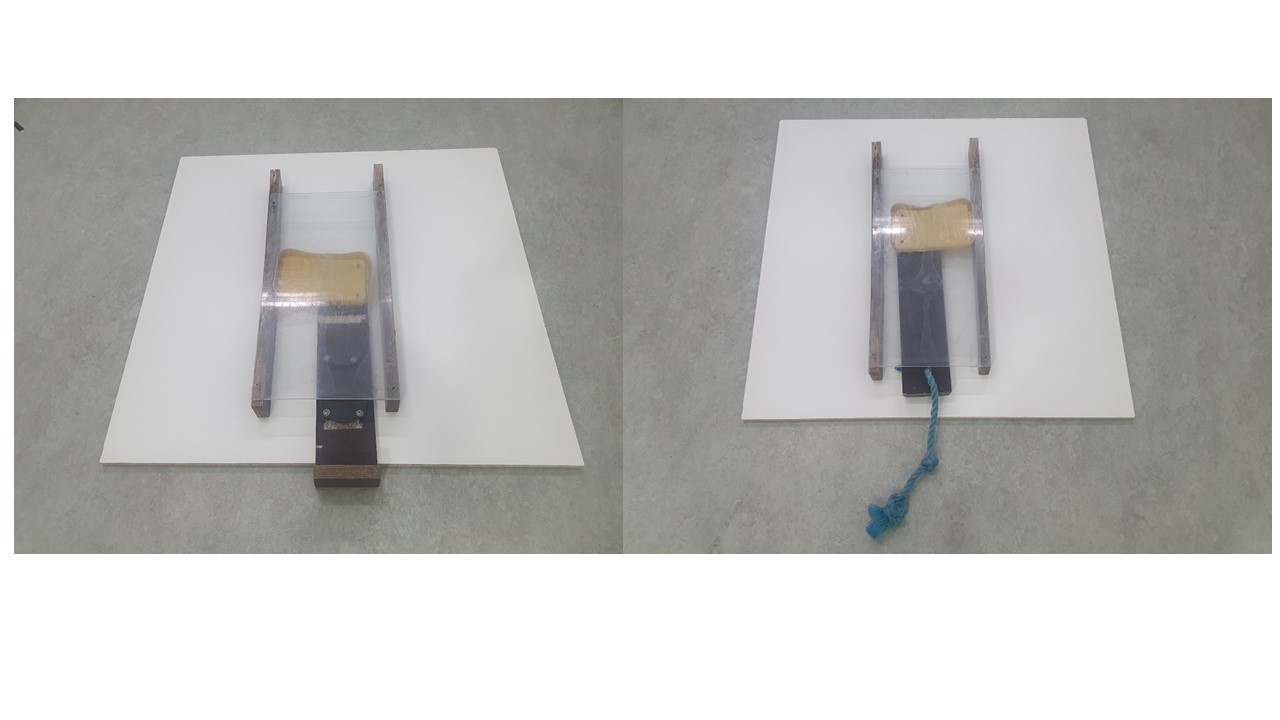
**

SI Fig. 1: Pictures of the training apparatuses. A drawer that dogs could pull with the paw and obtain food from the yellow container (left) and a rope that dogs could pull with the mouth and obtain food from the yellow container (right).

**Food preference test**

As dogs have problems with quantity discrimination (Range et. al, 2014), we used quality differences for the outcomes of the stag hunt game. We experimentally tested for individual food preferences using a food preference test (Brucks et al., 2016). The dog started sitting next to the owner while the experimenter baited a plate with one piece of kibble with her back turned to the dog. The experimenter showed the plate to the dog and allowed them to smell it before placing the plate on the floor 60cm away from the dog. The experimenter then said “okay” and allowed the dog to approach. If the dog ate the food, the experimenter picked up the plate and baited it again. If the dog ate the food six times in a row, this was established as a palatable LVR If the dog did not eat the kibble, a new food would be tested. However, all dogs ate the kibble.

For the second part, the dog sat by the owner again while the experimenter baited one plate with kibble and the other with a food the owner informed was preferred by the dog. The options were sausage, cheese, or commercial treats brought by the owner. The experimenter showed both plates to the dog and allowed it to smell them before placing them on the floor 60cm away from the dog and 60cm cm apart from each other. The experimenter then said “okay” and allowed the dog to move freely. After the dog ate one food, the other was immediately removed. This was repeated 12 times, alternating the position of each food (right of left) between trials. When the dog chose the new food at least nine times, that food was considered the HVR. If the dog chose the new food eight times or less, we repeated the procedure with a different type of food (details in the supplementary material). If the dog never formed a preference, which was the case for three dogs, they were included in the control group.

SI Table 1. Participants and their characteristics.

| Dog | Sex | Age | Breed | Group | Task learned | High value reward | Low value reward |
| --- | --- | --- | --- | --- | --- | --- | --- |
| Fanny | Female | 2 | Standard Poodle | Typical payoff | Rope | Cheesy sausage | Kibble |
| Sepp | Male | 10 | Beagle | Typical payoff | Rope | Sausage | Kibble |
| Marley | Male | 12 | Mixed breed | Typical payoff | Drawer | Cheese | Kibble |
| Ona | Female | 2 | Czechoslovakian Wolfdog | Typical payoff | Drawer | Cheese | Kibble |
| Loki | Male | 7 | Australian Shepherd | Typical payoff | Drawer | Sausage | Kibble |
| Tali | Female | 5 | American Staffordshire Terrier | Typical payoff | Drawer | Cheesy sausage | Kibble |
| Sahibu | Male | 8 | Mixed breed | Typical payoff | Drawer | Cheesy sausage | Kibble |
| Simba | Male | 3 | Border Collie | Typical payoff | Drawer | Sausage | Kibble |
| Albi | Male | 9 | Czechoslovakian Wolfdog | Typical payoff | Drawer | Sausage | Kibble |
| Blue | Female | 8 | Australian Shepherd | Typical payoff | Drawer | Sausage | Kibble |
| Kiba | Female | 6 | German Shepherd | Same reward | Drawer | Sausage | Sausage |
| Gaia | Female | 5 | Portuguese Water Dog | Same reward | Drawer | Sausage | Sausage |
| Emil | Male | 2 | Standard Poodle | Same reward | Drawer | Sausage | Sausage |
| Filou | Male | 1 | Mixed breed | Same reward | Drawer | Sausage | Sausage |
| Lia | Female | 3 | Jack Russel Terrier | Same reward | Drawer | Sausage | Sausage |
| Wilson | Male | 2 | Mixed breed | Same reward | Drawer | Sausage | Sausage |
| Yukibo | Female | 2 | Spitz | Same reward | Drawer | Sausage | Sausage |
| Jamie | Male | 4 | Greyhound | Same reward | Drawer | Sausage | Sausage |
| Moon | Female | 1 | Australian Shepherd | Same reward | Drawer | Sausage | Sausage |

**Model stability**

SI Table 2. Results of subject’s choice model including model stability and confidence intervals.

|  |  |  |  |  |  | Model stability | | Confidence interval | |
| --- | --- | --- | --- | --- | --- | --- | --- | --- | --- |
| Term | Estimate | SE | X² | df | P | Min | Max | Lower | Upper |
| Intercept | -2.537 | 0.436 | - | - | - | -2.880 | -2.065 | -3.391 | 1.683 |
| **Group (1)** | 1.303 | 0.482 | 6.543 | 1 | 0.011 | 0.955 | 2.074 | 0.358 | 2.249 |
| **Partner choice (1)** | 1.049 | 0.259 | 17.244 | 1 | <0.001 | 0.872 | 1.219 | 0.541 | 1.557 |
| **Trial (2)** | -0.179 | 0.089 | 4.135 | 1 | 0.042 | -0.255 | -0.085 | -0.353 | -0.004 |
| Session (2) | -0.701 | 0.249 | 13.806 | 1 | <0.001 | -0.869 | -0.489 | -1.189 | -0.212 |
| Age (2) | -0.579 | 0.257 | 11.311 | 1 | <0.001 | -0.985 | -0.306 | -1.082 | -0.076 |
| Side (1) | - | - | 3.319 | 2 | 0.190 | -0.325 | -0.201 | -0.583 | 0.263 |
| Distance (1) | 0.039 | 0.123 | 0.095 | 1 | 0.757 | -0.009 | 0.110 | -0.203 | 0.280 |

**
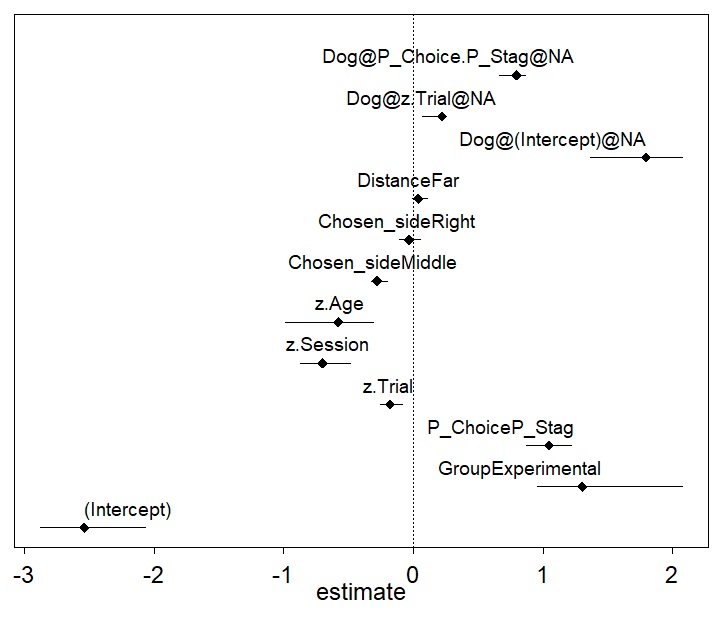
**

SI Fig. 2: Model stability of subject’s choice model

SI Table 3. Results of matching choices model including model stability and confidence intervals.

|  |  |  |  |  |  | Model stability | | Confidence interval | |
| --- | --- | --- | --- | --- | --- | --- | --- | --- | --- |
| Term | Estimate | SE | X² | df | P | Min | Max | Lower | Upper |
| Intercept | 1.884 | 0.424 | - | - | - | 1.292 | 2.146 | 1.053 | 2.714 |
| Trial (1) | 0.055 | 0.084 | 0.433 | 1 | 0.510 | 0.011 | 0.106 | -0.109 | 0.219 |
| Session (1) | -0.026 | 0.094 | 0.076 | 1 | 0.783 | -0.106 | 0.011 | -0.211 | 0.159 |
| Age (1) | 0.257 | 0.117 | 4.099 | 1 | 0.043 | 0.155 | 0.347 | 0.028 | 0.486 |
| Side (2) | 0.03 | 0.146 | 4.508 | 2 | 0.105 | -0.199 | 0.326 | -0.424 | 0.548 |
| Distance (2) | - | - | 0.041 | 1 | 0.840 | -0.079 | 0.096 | -0.256 | 0.316 |
| **Group*Condition (2)** | - | - | 8.698 | 2 | 0.013 | 0.930 | 2.479 | 0.258 | 3.952 |

**
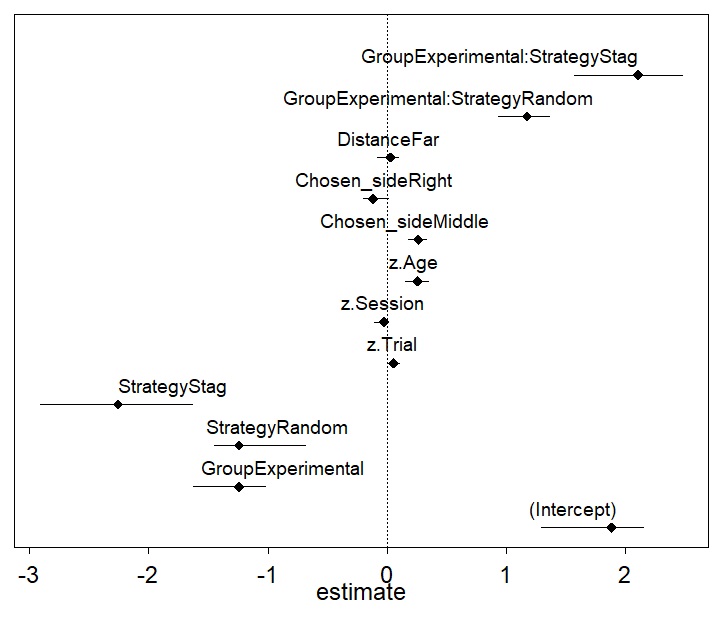
**

SI Fig. 3: Model stability of condition model

**Reliability coding**

A second person coded 20% of the videos and we analyzed interobserver reliability with Cohen’s kappa, using the package “irr” in R. Kappa values are shown in SI Table 2.

SI Table 4. Kappa’s value for each variable.

| **Variable** | **Kappa** |
| --- | --- |
| Subject choice | 0.931 |
| Side | 0.773 |
| Distance | 0.822 |
| Success | 0.952 |
| Match | 0.947 |

Fanny (experimental group)


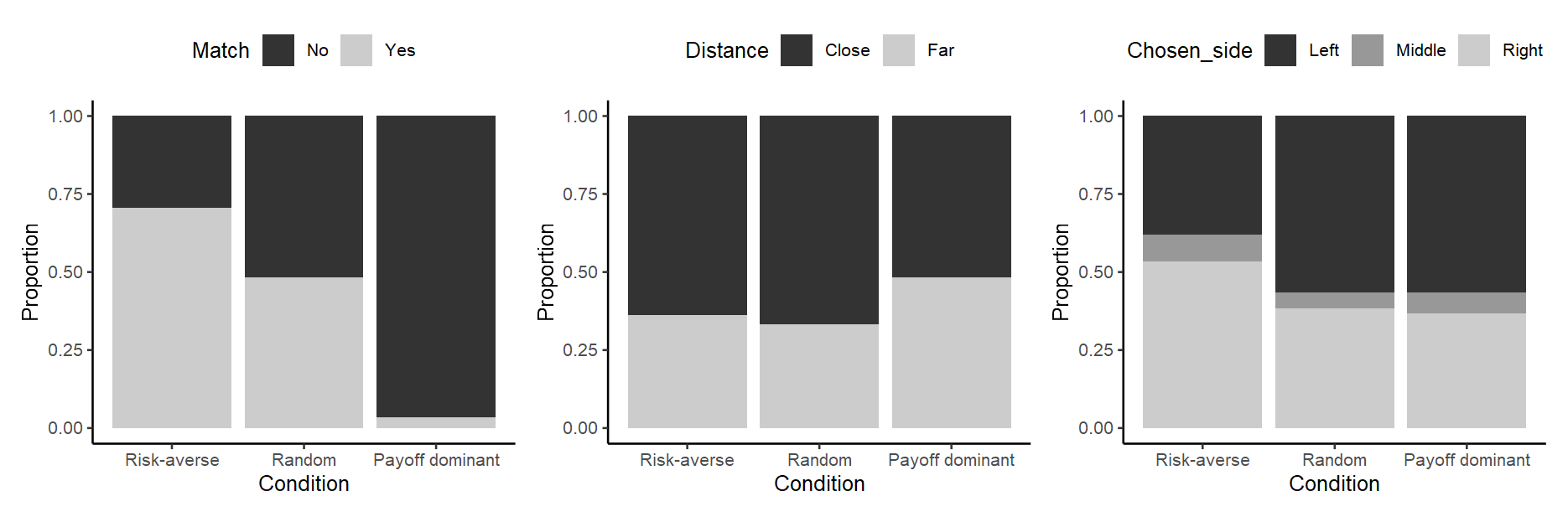


Sepp (experimental group)


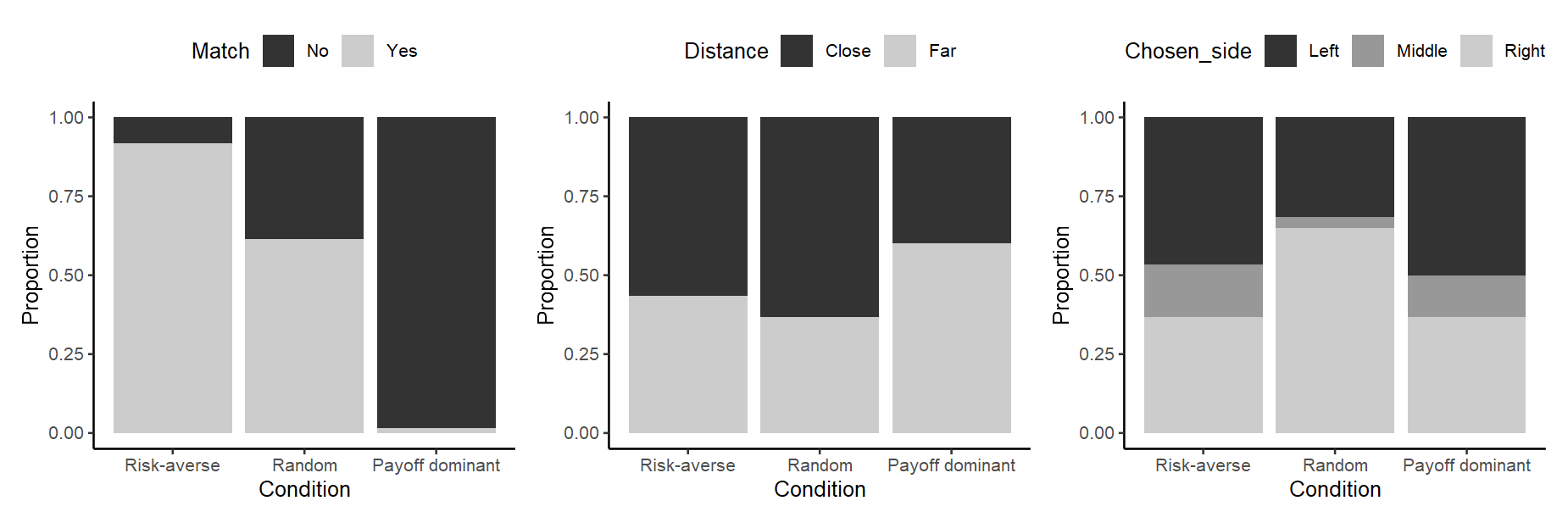


Marley (experimental group)


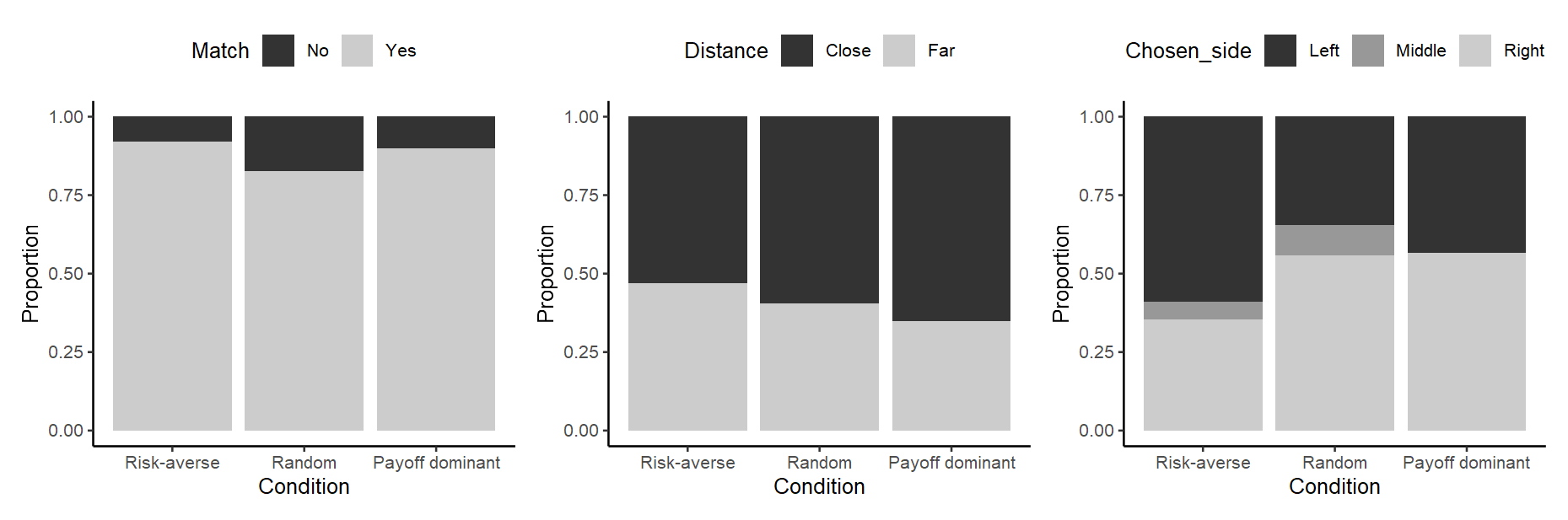


Ona (experimental group)


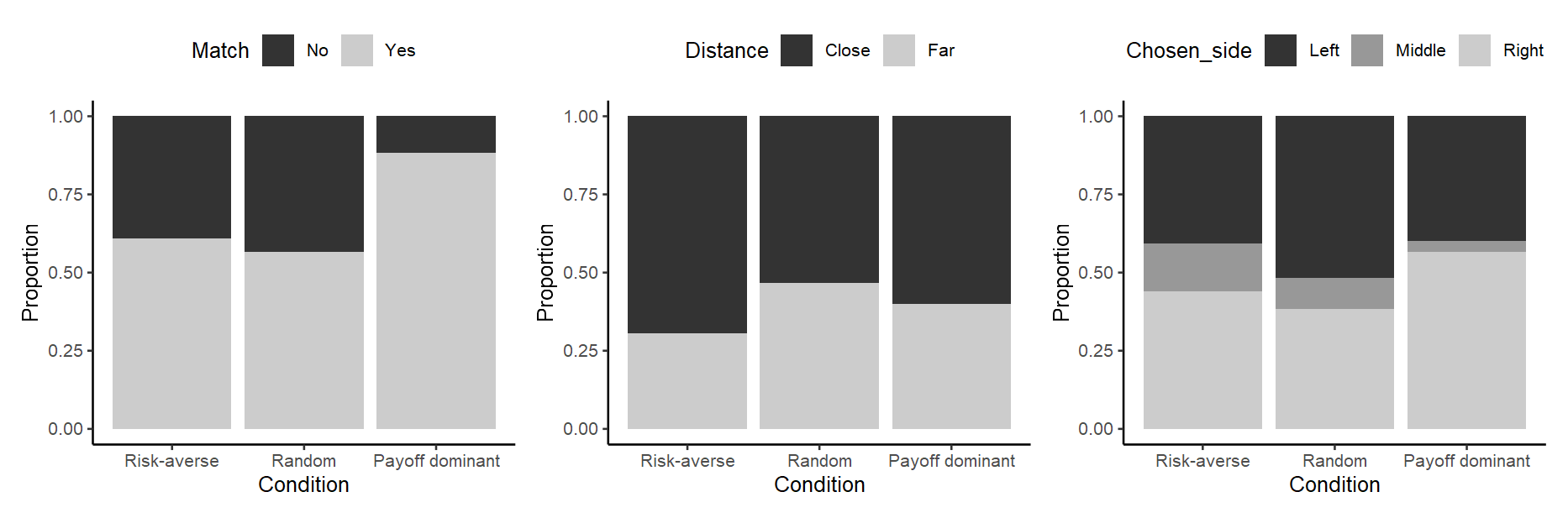


Loki (experimental group)


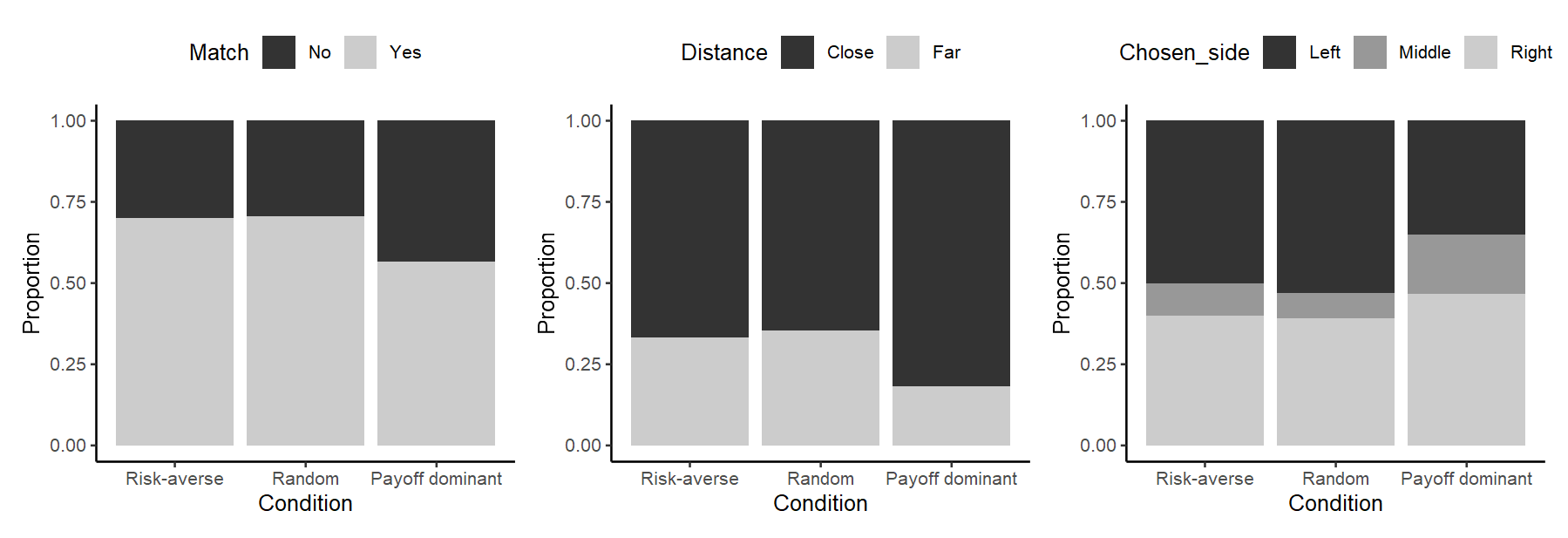


Tali (experimental group)


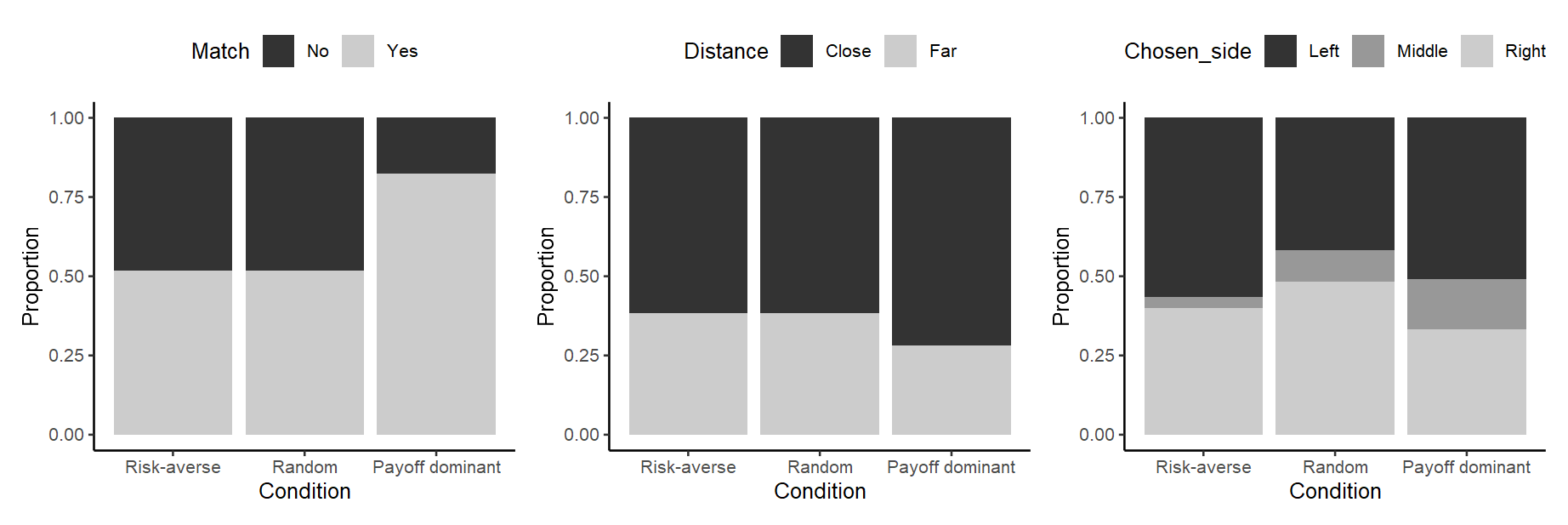


Sahibu (experimental group)


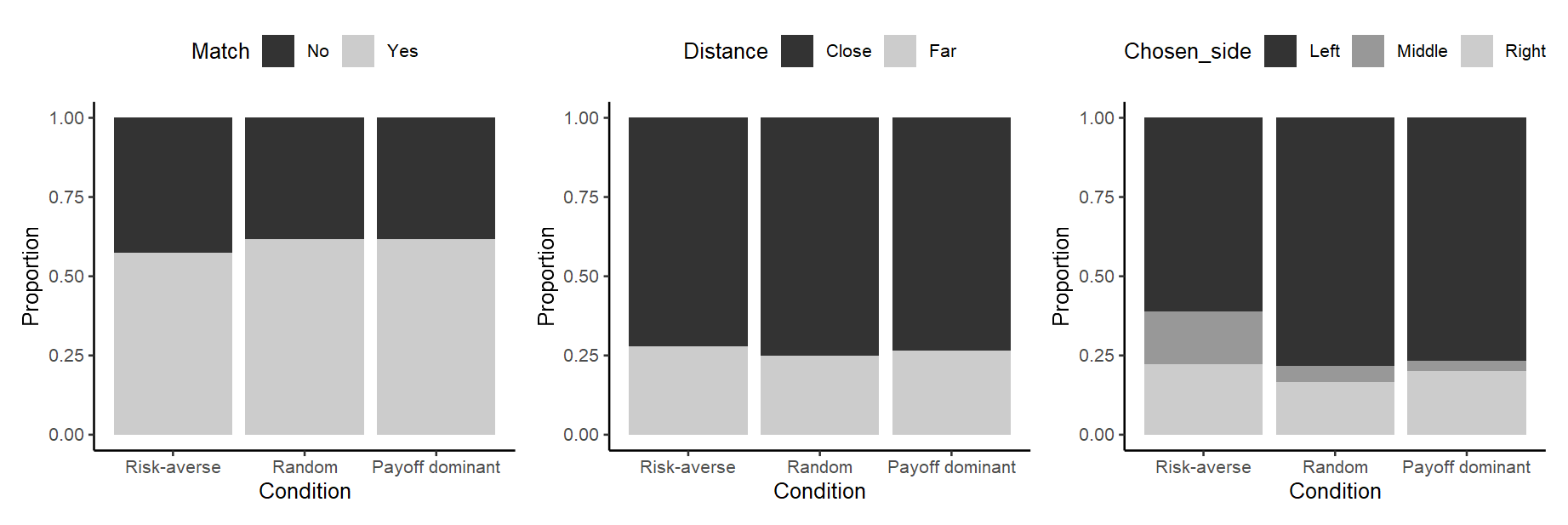


Simba (experimental group)


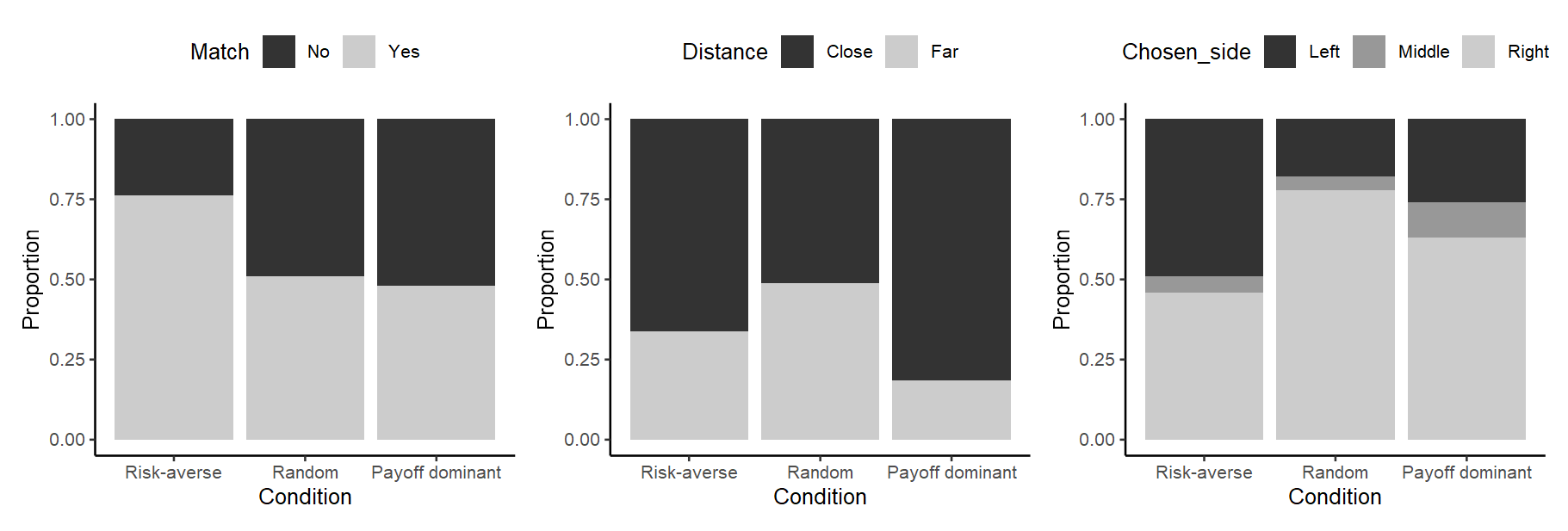


Albi (experimental group)


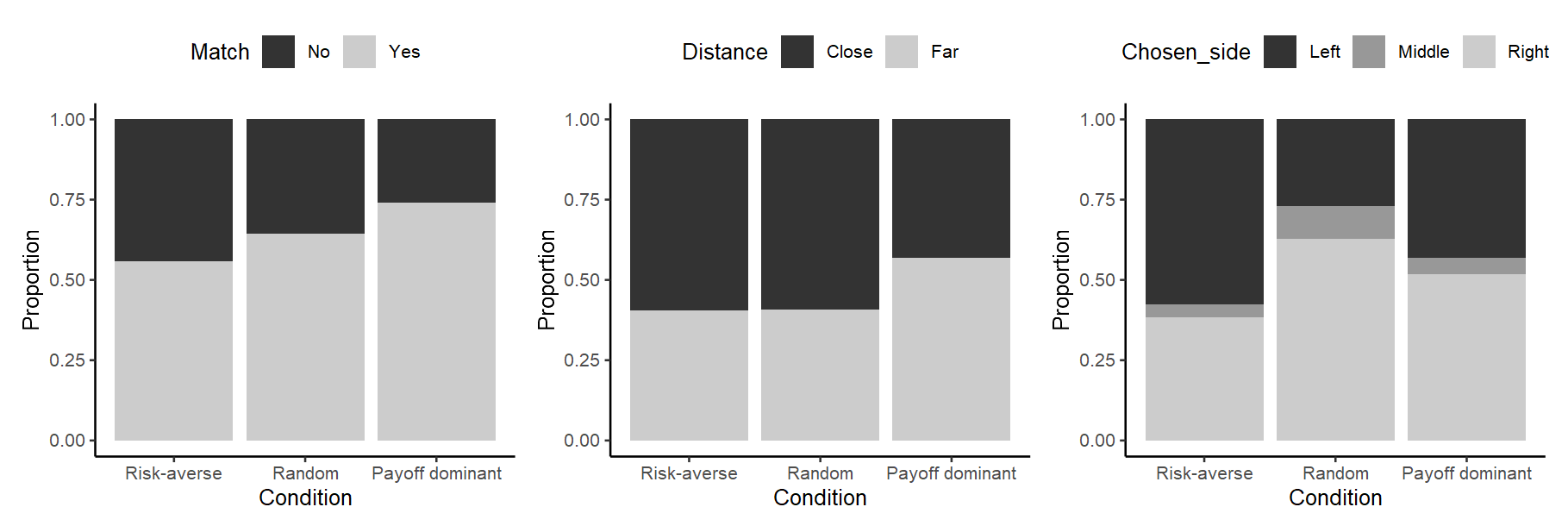


Blue (experimental group)


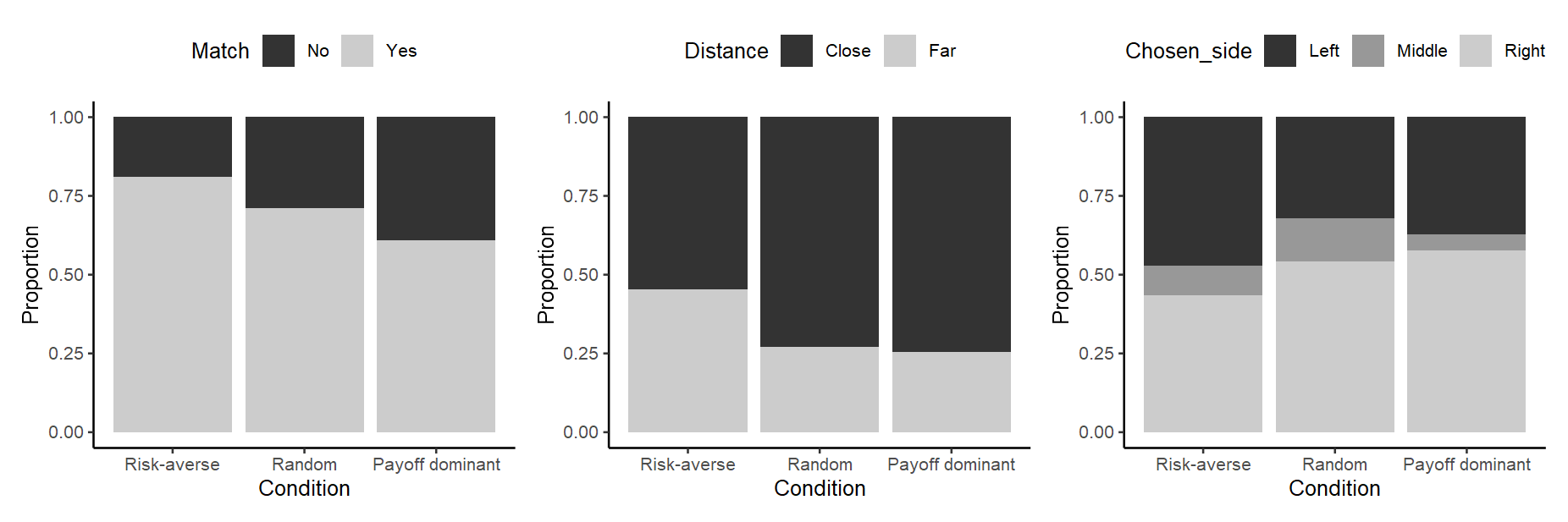


Kiba (control group)


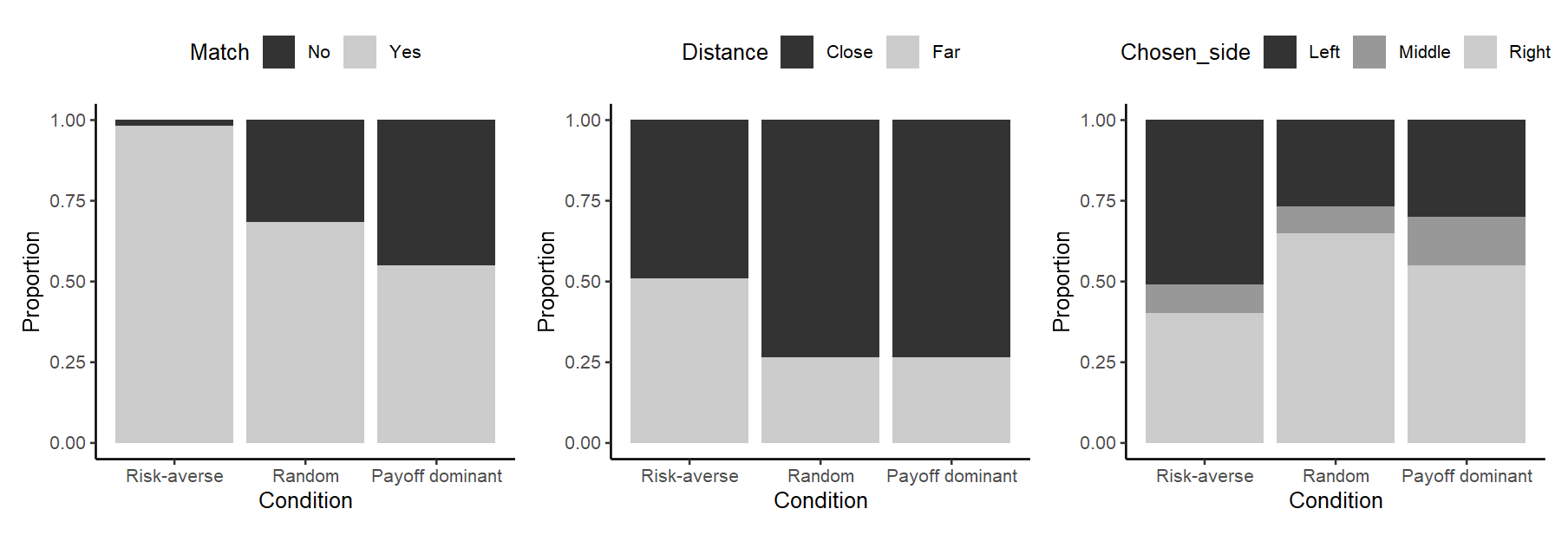


Gaia (control group)


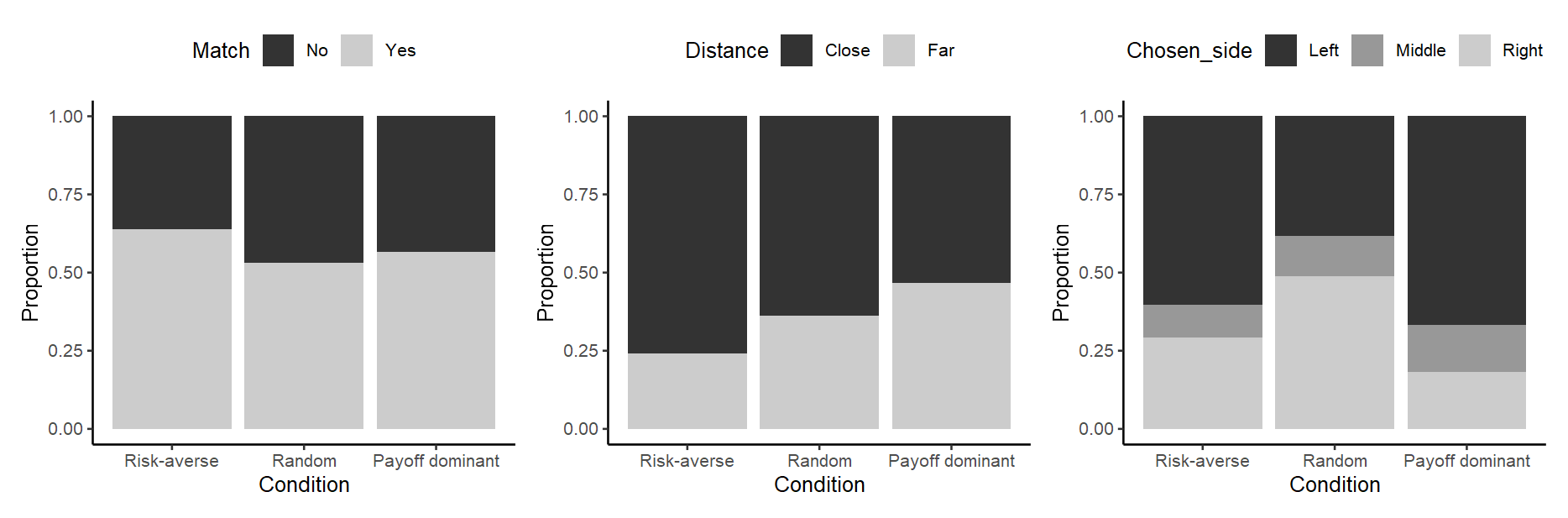


Emil (control group)


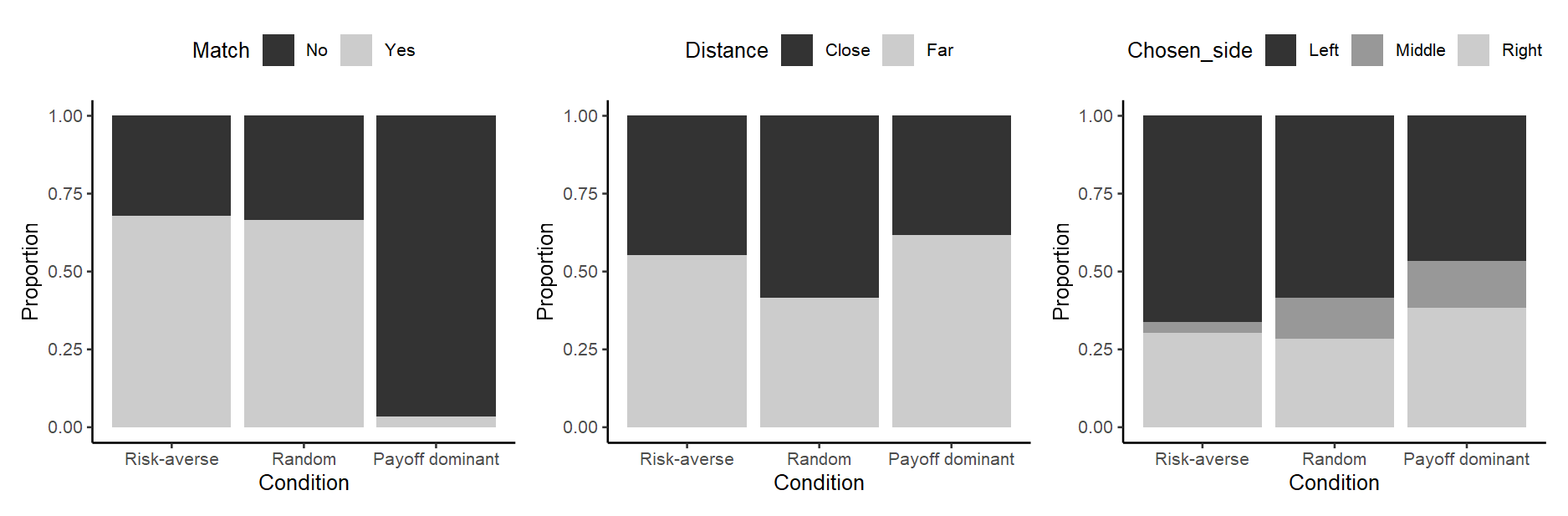


Filou (control group)


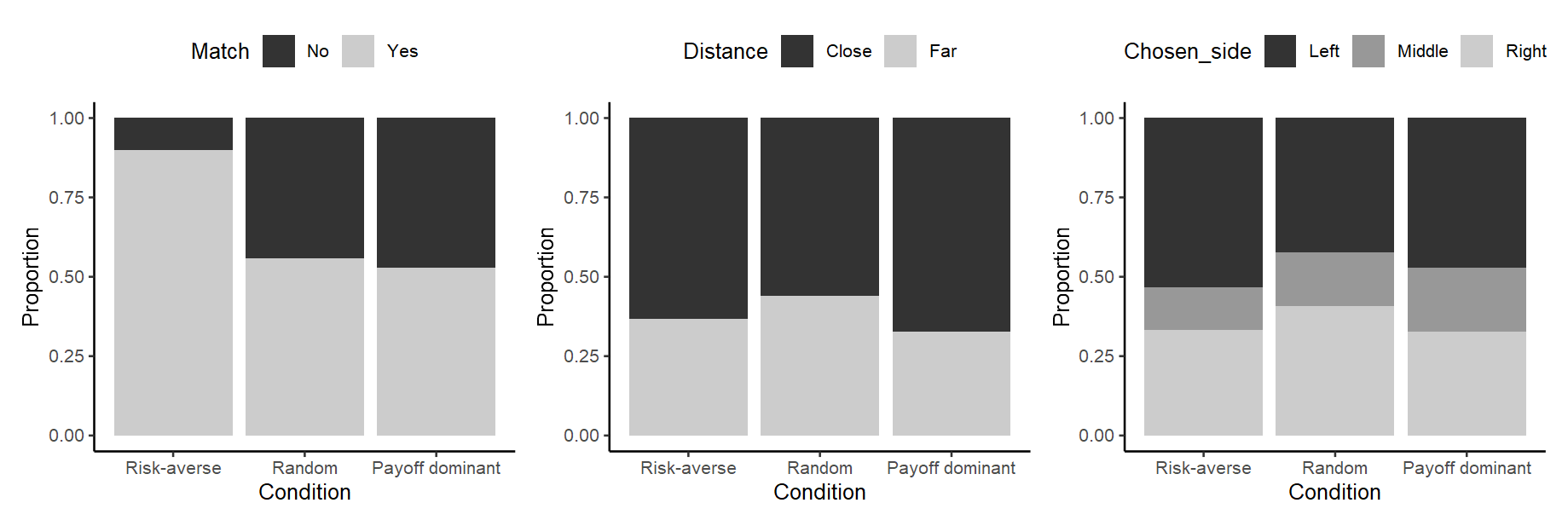


Lia (control group)


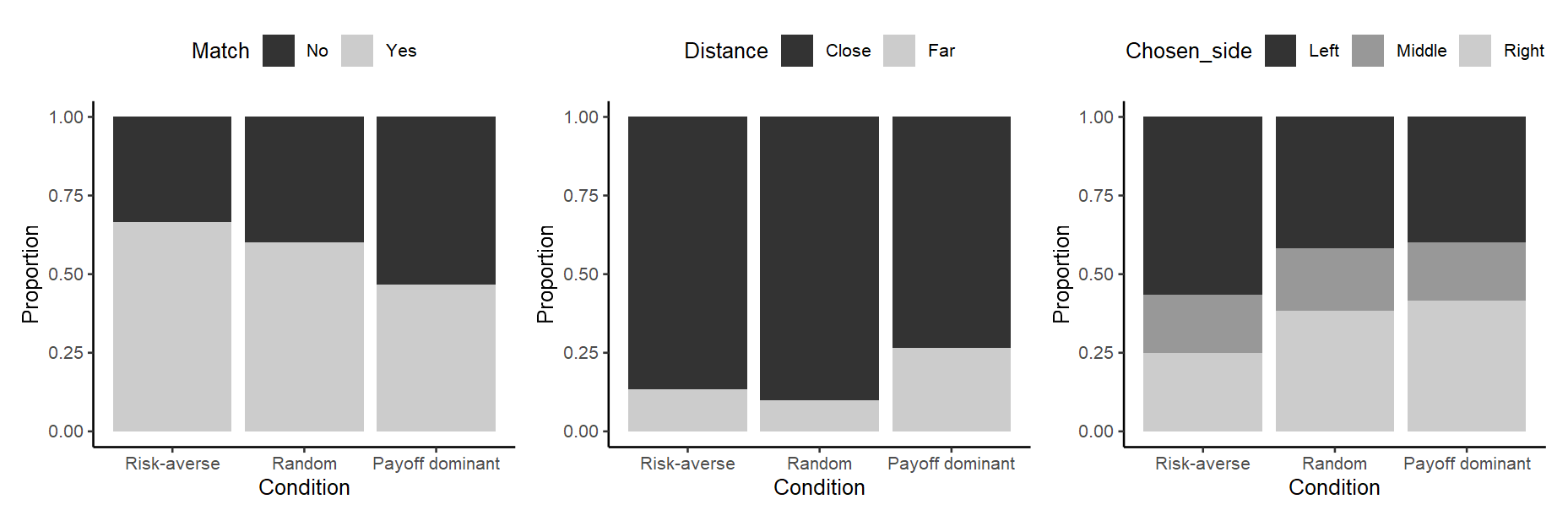


Wilson (control group)


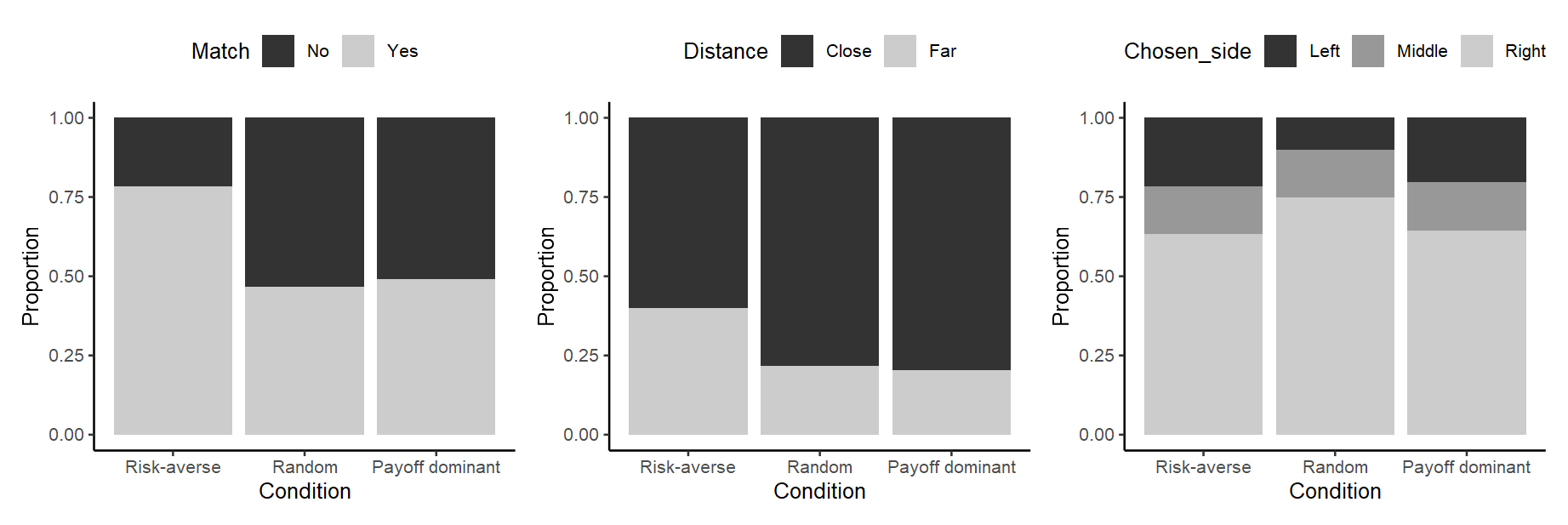


Yukibo (control group)


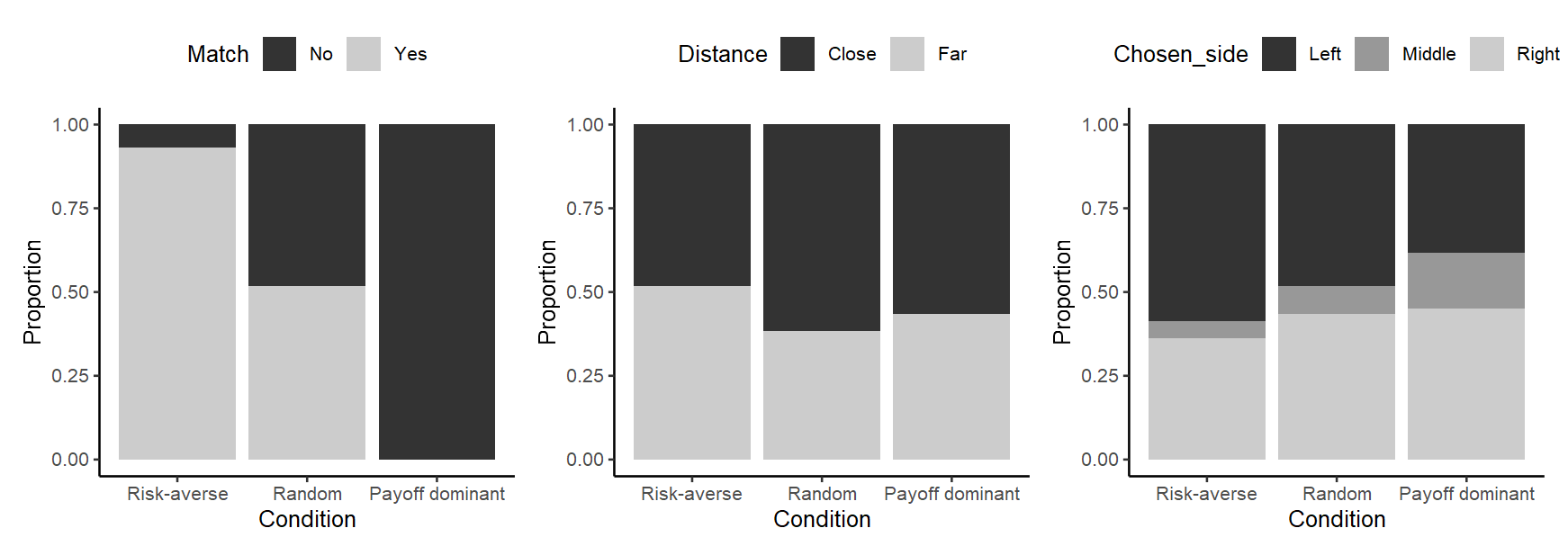


Jamie (control group)


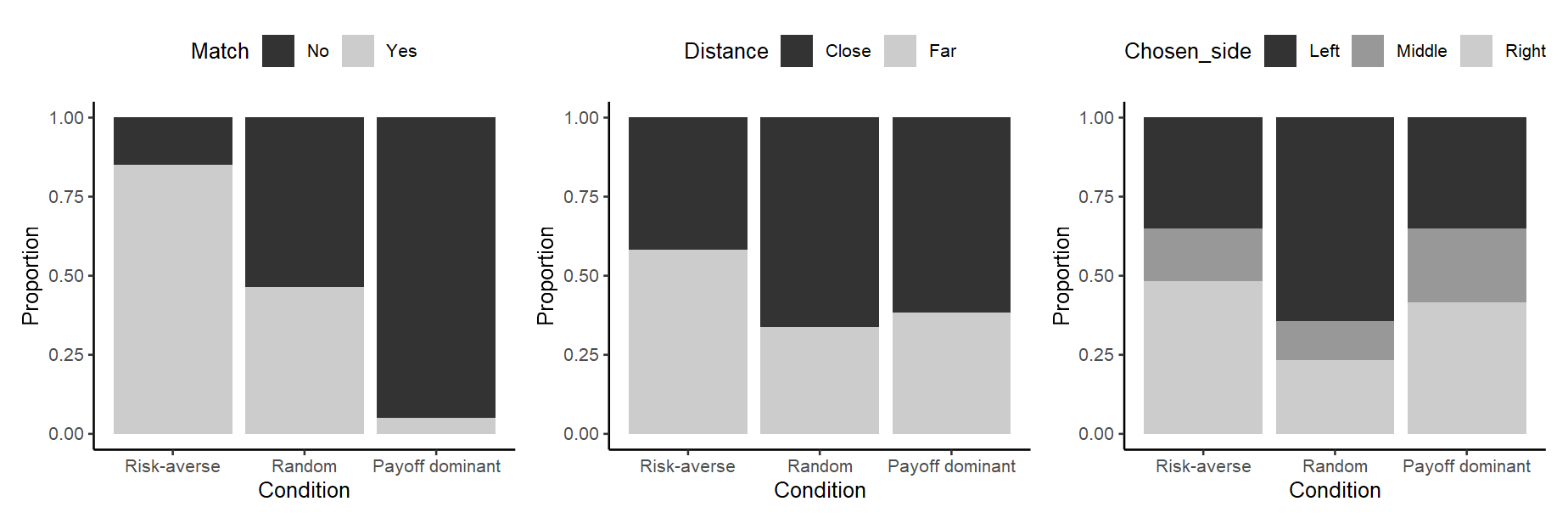


Moon (control group)


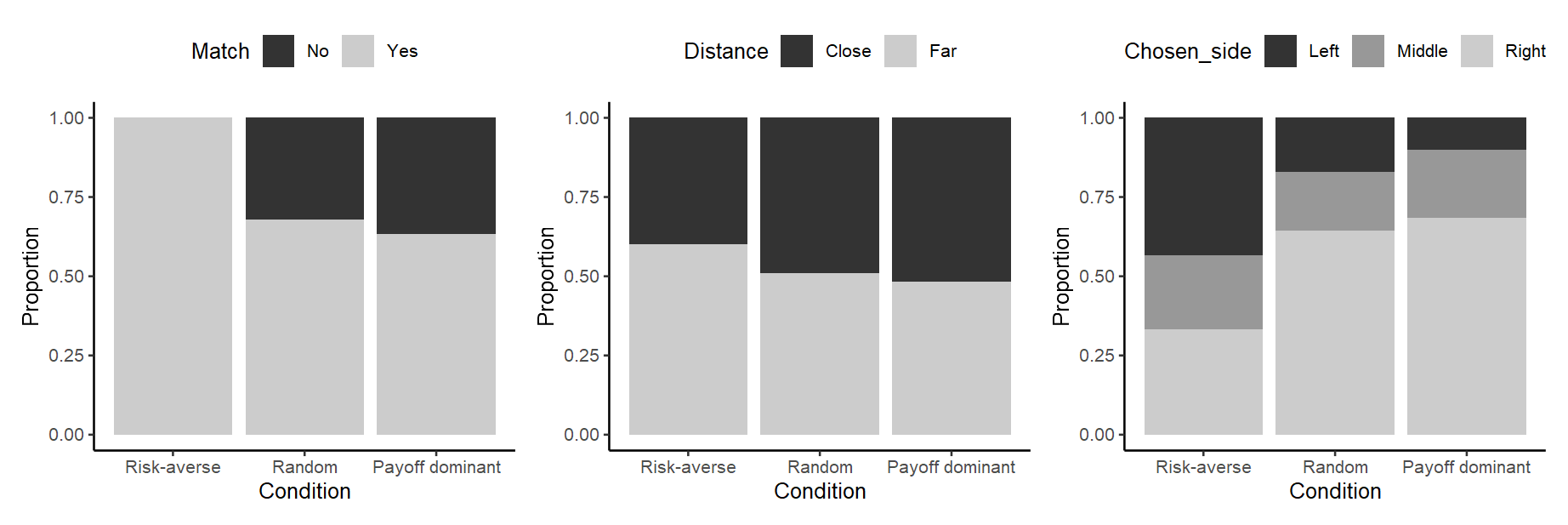


SI Fig. 3: plots of individual dogs’ choices. Left: proportion of matching in different conditions. Middle: proportion of choosing the close of far apparatus. Right: proportion of choosing the apparatus on the left, middle or right.

**SI references**

Brucks, D., Essler, J. L., Marshall-Pescini, S., & Range, F. (2016). Inequity aversion negatively affects tolerance and contact-seeking behaviours towards partner and experimenter. *PLoS One*, *11*(4), e0153799.

Range, F., Jenikejew, J., Schröder, I., & Virányi, Z. (2014). Difference in quantity discrimination in dogs and wolves. *Frontiers in Psychology*, *5*, 105702.
